# Characterization of ginsenosides from *Panax japonicus* var. *major* (Zhu-Zi-Shen) based on ultra-high performance liquid chromatography/quadrupole time-of-flight mass spectrometry and desorption electrospray ionization-mass spectrometry imaging

**DOI:** 10.1186/s13020-023-00830-9

**Published:** 2023-09-08

**Authors:** Meiting Jiang, Xiaohang Li, Yuying Zhao, Yadan Zou, Maoli Bai, Zhiming Yang, Wei Wang, Xiaoyan Xu, Hongda Wang, Wenzhi Yang, Qinhua Chen, Dean Guo

**Affiliations:** 1 National Key Laboratory of Chinese Medicine Modernization, State Key Laboratory of Component-based Chinese Medicine, Tianjin University of Traditional Chinese Medicine, 10 Poyanghu Road, Tianjin, 301617 China; 2 Haihe Laboratory of Modern Chinese Medicine, Tianjin University of Traditional Chinese Medicine, 10 Poyanghu Road, Tianjin, 301617 China; 3 Shenzhen Baoan Authentic TCM Therapy Hospital, Shenzhen, 518101 China; 4 National Engineering Laboratory for TCM Standardization Technology, Shanghai Research Center for Modernization of Traditional Chinese Medicine, Shanghai Institute of Materia Medica, Chinese Academy of Sciences, 501 Haike Road, Shanghai, 201203 China

**Keywords:** *Panax japonicus* var. *major*, Ginsenoside, UHPLC/QTOF-MS, DESI-MSI, Precursor ions list, Spatial distribution

## Abstract

**Background:**

*Panax japonicus* var. *major* (PJM) belongs to the well-known ginseng species used in west China for hundreds of years, which has the effects of lung tonifying and *yin* nourishing, and exerts the analgesic, antitussive, and hemostatic activities. Compared with the other *Panax* species, the chemical composition and the spatial tissue distribution of the bioactive ginsenosides in PJM have seldom been investigated.

**Methods:**

Ultra-high performance liquid chromatography/quadrupole time-of-flight mass spectrometry (UHPLC/QTOF-MS) and desorption electrospray ionization-mass spectrometry imaging (DESI-MSI) were integrated for the systematic characterization and spatial tissue distribution studies of ginsenosides in the rhizome of PJM. Considering the great difficulty in exposing the minor saponins, apart from the conventional Auto MS/MS (**M1**), two different precursor ions list-including data-dependent acquisition (PIL-DDA) approaches, involving the direct input of an in-house library containing 579 known ginsenosides (**M2**) and the inclusion of the target precursors screened from the MS^1^ data by mass defect filtering (**M3**), were developed. The in situ spatial distribution of various ginsenosides in PJM was profiled based on DESI-MSI with a mass range of *m/z* 100–1500 in the negative ion mode, with the imaging data processed by the High Definition Imaging (HDI) software.

**Results:**

Under the optimized condition, 272 ginsenosides were identified or tentatively characterized, and 138 thereof were possibly not ever reported from the *Panax* genus. They were composed by 75 oleanolic acid type, 22 protopanaxadiol type, 52 protopanaxatriol type, 16 octillol type, 19 malonylated, 35 C-17 side-chain varied, and 53 others. In addition, the DESI-MSI experiment unveiled the differentiated distribution of saponins, but the main location in the cork layer and phloem of the rhizome. The abundance of the oleanolic acid ginsenosides was high in the rhizome slice of PJM, which was consistent with the results obtained by UHPLC/QTOF-MS.

**Conclusion:**

Comprehensive characterization of the ginsenosides in the rhizome of PJM was achieved, with a large amount of unknown structures unveiled primarily. We, for the first time, reported the spatial tissue distribution of different subtypes of ginsenosides in the rhizome slice of PJM. These results can benefit the quality control and further development of PJM and the other ginseng species.

**Supplementary Information:**

The online version contains supplementary material available at 10.1186/s13020-023-00830-9.

## Background

The genus *Panax* L. (Araliaceae) has a long history of utilization, particularly in China and the other Asian nations. Due to the tonifying effects on human bodies, ginseng is often used as a medicinal and edible tonic [1, 2]. The *Panax* genus involves multiple different species worldwide, among which *P. ginseng* C. A. Meyer (Ren-Shen), *P. quinquefolius* L. (Xi-Yang-Shen), *P. notoginseng* (Burk.) F. H. Chen (San-Qi), *P. japonicus* C. A. Meyer (Zhu-Jie-Shen), and *P. japonicus* C. A. Mey. var. *major* (Burk.) C. Y. Wu et K. M. Feng (Zhu-Zi-Shen), are well known [3]. The chemical components contained in the *Panax* species include the saponins (also known as ginsenosides), polysaccharides, flavonoids, sterols, and volatile oils, etc. [3–5]. In particular, the ginsenosides are the most common and most important active substances in diverse *Panax*-derived herbal medicines and products [1, 4]. Generally, they are used as the quality control markers for the drug materials of ginseng and the prescription preparations that contain ginseng. In structure, a ginsenoside is an oligosaccharide glycoside of either the dammarane or the oleanane-type triterpenoid skeleton [6]. As a result of the structure difference on the sapogenins, the known ginsenosides can be divided into multiple subtypes, and the protopanaxadiol (PPD) and protopanaxatriol (PPT) types thereof are the most important. Notably, *P. japonicus* var. *major* (PJM) is a famous ginseng species used in west China for hundreds of years [7], and has the effects of lung tonifying, *yin* nourishing, analgesic, antitussive, and hemostatic activities [8, 9]. Different from the other ginseng species, the oleanolic acid-type (OA) saponins, such as ginsenoside Ro, chikusetsusaponins IVa, -IV, are the main saponin components in PJM [10, 11]. According to a systematic literature review, the researches regarding the characterization of ginsenosides in PJM are rare, as a whole. A previous study, by integrating two different liquid chromatography–mass spectrometry (LC–MS) techniques, could identify or tentatively characterize 78 compounds in PJM [12]. Another study utilized the LC–MS method to analyze and characterize the metabolites of PJM and the other four ginseng species [13]. However, because of the covering of abundant components, to expose and identify the minor saponins from PJM is rather difficult necessitating the development of a more potent analytical approach. Additionally, the location of different subtypes of ginsenosides in the rhizome tissue of PJM remains unclear, hitherto.

Medicinal herbs have the potential for new drugs discovery. Systematic elucidation of the herbal components acts as a prerequisite prior to revealing the mechanism of action and formulating the quality control standards. Chromatography methods, such as thin layer chromatography (TLC), high-performance liquid chromatography (HPLC), gas chromatography (GC), ion chromatography (IC), and LC–MS, are commonly employed for this purpose [14–16]. In particular, LC–MS integrates the powerful separation capability of LC and the high sensitivity and selectivity of MS, thus providing multiple dimensions of information beneficial to the rapid multicomponent characterization for herbal medicines [17, 18]. Furthermore, the well-developed multi-dimensional chromatography has covered the increasing application facilitating the in-depth characterization of herbal components [19, 20], because of the greatly expanded peak capacity and improved selectivity of chromatographic separation [21]. In addition, MS has been rapidly developing targeted to improve both the resolution and the separation dimension. Particularly, the high-resolution time-of-flight (TOF) and ultra-high resolution Orbitrap mass spectrometers can enable more reliable identification results [22]. Important progress in the improvement of sensitivity for various MS analyzers, such as the quadrupole, ion trap, TOF, and their combinations, has been comprehensively summarized in a review [23]. From the viewpoint of data acquisition, data-dependent acquisition (DDA) is more preferably utilized for the untargeted multicomponent characterization for the herbal medicines compared with the data-independent acquisition (DIA), due to the high quality in the MS^2^ or MS^n^ spectra and the easiness in data interpretation, but the coverage of DDA can be largely damaged when facing the complicated chemical matrix like an herbal extract. As a solution, the inclusion of a precursor ions list (PIL) in DDA can remarkably improve the coverage on the components of interest [24, 25].

Mass spectrometry imaging (MSI) enables in situ visualization of the spatial distribution of molecules without the prior labelling [26–28]. Currently, matrix-assisted laser desorption ionization (MALDI) and desorption electrospray ionization (DESI) are the most common ionization techniques used in MSI [29]. In contrast, the MALDI-MSI technique involves matrix coating, which may introduce the excessive noise peaks [30]. DESI-MSI, as an ambient imaging technique, allows the desorption and ionization of the analytes directly from the sample surface with the minimal sample pretreatment. This technique was first used for directly visualizing the spatial localization of biological samples in 2006 [31], and currently DESI-MSI is applied in various research fields, such as cancer [32], oncology [33], functional food [34], fingerprint recognition [35], and environmental protection, etc. [36]. The plant biologists have utilized MSI in a broad range of areas, such as the primary metabolism, plant defense, plant lipids, natural products, and the developing field of spatial metabolomics [37]. For example, the locations of various metabolites in *Gelsemium elegans* and the raw/processed Fuzi were mapped by using DESI-MSI [38, 39]. Interestingly, the use of DESI-MSI in analyzing the saponins in *Panax* species can offer more elaborate tissue distribution information, compared with LC–MS. The location of saponins and characteristic chemical markers in the roots of *P. quinquefolius* and *P. notoginseng* were visualized, which demonstrated a specific distribution pattern and content variation within the different plant tissues [40]. LC–MS/MS in combination with nano-DESI was utilized to explore the distribution of ginsenoside Rg1 in different tissues, especially in the kidney and brain [41]. The integration of DESI-MSI and UHPLC/QTOF-MS was powerful in discriminating different parts and ages of ginseng [42].

**Fig. 1:**
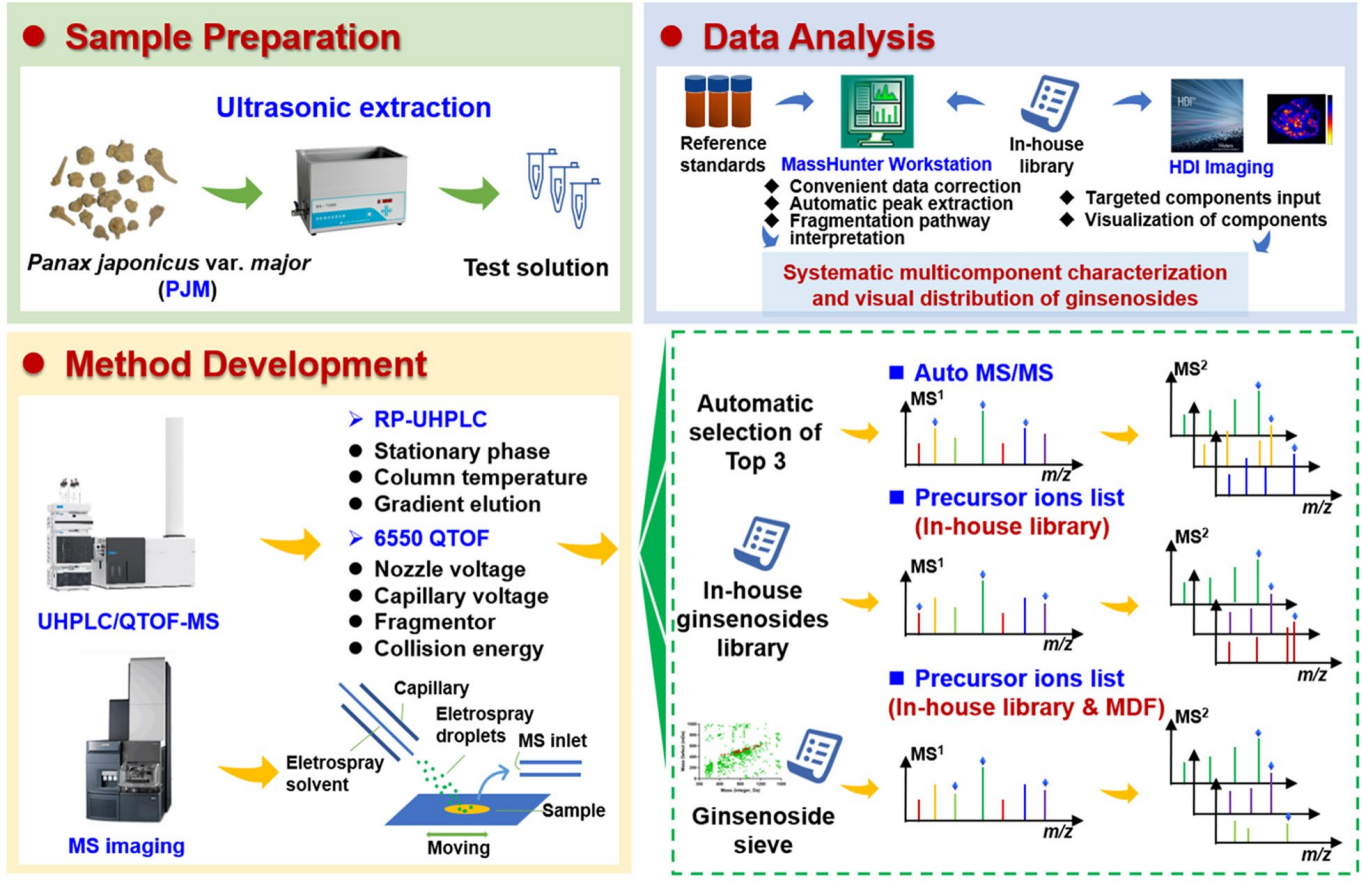
General technical roadmap for the comprehensive characterization and spatial tissue distribution of ginsenosides in the rhizome of *Panax japonicus* var. *major* (PJM) by integrating UHPLC/QTOF-MS and DESI-MSI

The aim of this work was to comprehensively characterize the ginsenosides from the rhizome of PJM, and locate different subtypes of ginsenosides in the rhizome tissue, by integrating UHPLC/QTOF-MS and DESI-MSI analyses. Figure 1 presents the conceptual framework involved in this work. In particular, to identify more minor ginsenosides from PJM, several aspects of efforts had been made to elevate the performance of UHPLC/QTOF-MS: (1) systematic optimization of both the reversed-phase UHPLC separation and the MS detection using 6550 QTOF in the negative mode; (2) aside from the conventional Auto MS/MS (namely **M1**), new development of another two PIL-including DDA approaches (PIL-DDA), including the direct input of an in-house library containing 579 known ginsenosides (**M2**) and the inclusion of the target precursors screened from the MS^1^ data by mass defect filtering (MDF; **M3**); (3) searching of the in-house ginsenoside database and referring to 92 ginsenoside standards. By these efforts, the composition of ginsenosides in PJM was comprehensively depicted, and the spatial tissue distribution of different subtypes of ginsenosides in the rhizome tissue was mapped.

## Materials and methods

### Chemicals and materials

**Fig. 2:**
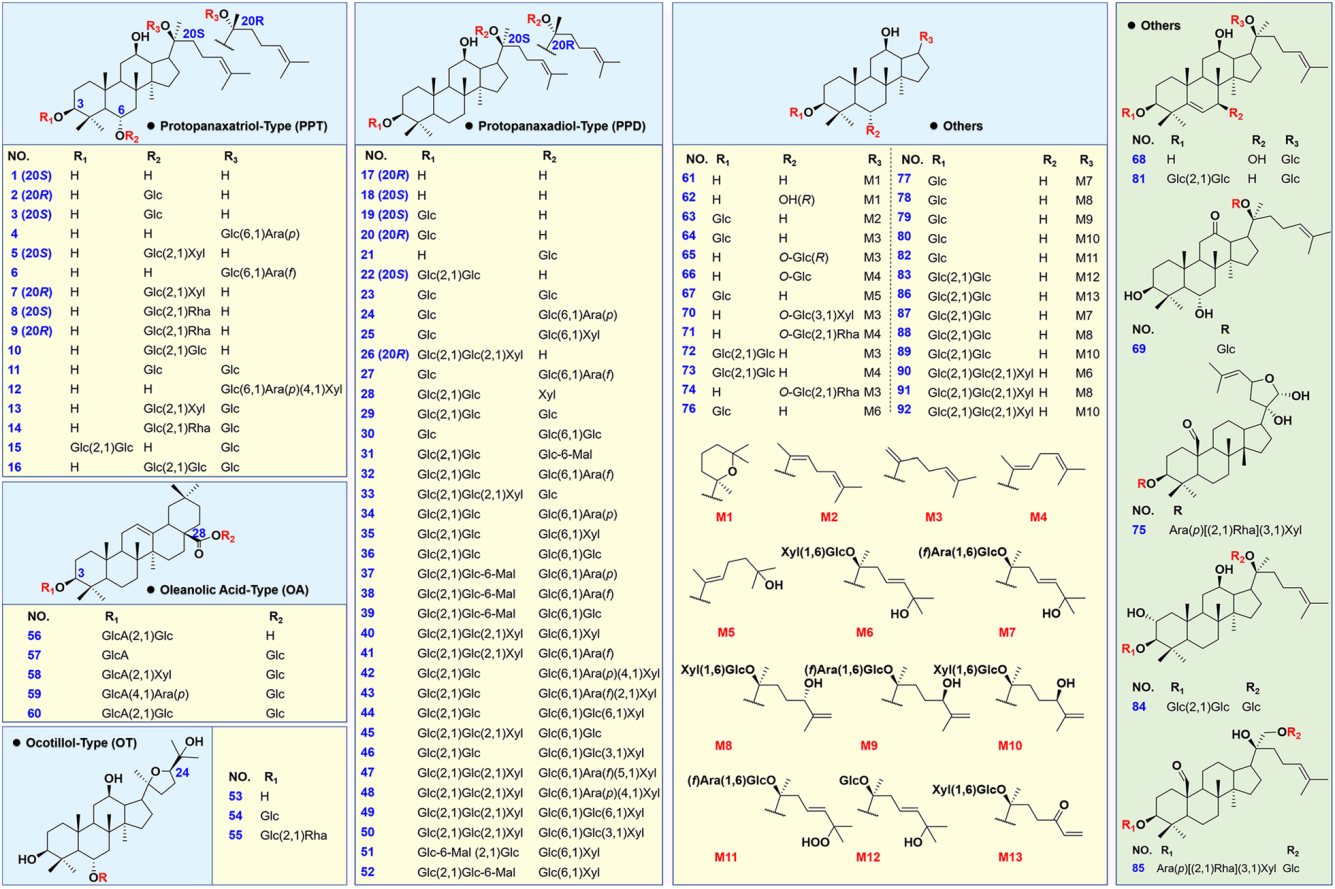
Chemical structures for 92 ginsenoside reference standards

In total, 92 ginsenosides were used as reference standards, which were either purchased from Shanghai Standard Biotech. Co., LTD. (Shanghai, China) or isolated by the authors from the *P*. *ginseng* and *P*. *notoginseng* roots. The chemical structures of these compounds and detailed information are shown in Fig. 2 and Additional file 1: Table S1. The raw materials of PJM were purchased from Hanzhong (Shaanxi, China), and authenticated by Prof. Xian-kuan Li (Tianjin University of Tradition Chinese Medicine). The voucher specimens were deposited at the authors’ laboratory in Tianjin University of Traditional Chinese Medicine (Tianjin, China). Both acetonitrile and formic acid (Fisher, Fair Lawn, NJ, USA) were the LC–MS grade. Ultra-pure water (18.2 MΩ·cm at 25 °C) was in-house prepared using a Milli-Q Integral 5 water purification system (Millipore, Bedford, MA, USA).

### Sample preparation

The PJM sample for the UHPLC/QTOF-MS analysis was prepared by an ultrasound-assisted extraction method [43]. In detail, approximately 500 mg of the fine powder was accurately weighed in a 15-mL centrifuge tube and extracted using 5 mL of 70% (v/v) methanol ultrasonically for 1 h (power: 400 W, frequency: 40 kHz). The lost weight was compensated with 70% (v/v) methanol. The extract was then centrifuged at 3219×*g* (equal to 4000 rpm) for 10 min, and the obtained supernatant was further transferred into a 10-mL volumetric flask. By adding 3 mL of 70% (v/v) methanol to the centrifuge tube, the extraction process was repeated. The supernatant pooled from these two extractions was diluted to a constant volume (10 mL). The test solution of PJM was diluted by fivefolds (concentration: 10 mg/mL of the drug material) and centrifuged at 11,481×*g* (14,000 rpm) for 10 min.

### Chromatography and mass spectrometry conditions

An Agilent 1290 Infinity II UHPLC system coupled with an Agilent 6550 QTOF mass spectrometer (Agilent Technologies, Santa Clara, CA, USA) was utilized to perform the multicomponent characterization of PJM. Chromatographic separation was performed on the ACQUITY UPLC BEH Shield RP18 column (2.1 × 100 mm, 1.8 μm), with the column temperature maintained at 30 °C. Gradient elution was conducted using a binary mobile phase (A: 0.1% formic acid in H_2_O; B: 0.1% formic acid in acetonitrile). The gradient elution program was as follows: 0–7 min, 15–22% (B); 7–11 min, 22% (B); 11–15 min, 22–29% (B); 15–20 min, 29–30% (B); 20–40 min, 30% (B); 40–42 min, 30–35% (B); 42–46 min, 35–42% (B); 46–56 min, 42–70% (B); 56–64 min, 70–95% (B); and 64–67 min, 95% (B). The flow rate was set at 0.3 mL/min. An injection volume of 3 µL was utilized for the analysis.

High-resolution MS spectra were recorded in the negative ESI (electrospray ionization) mode. The MS conditions were as the following: gas (N_2_) temperature, 200 °C; drying gas flow, 12 L/min; nebulizer pressure, 40 psi; fragmentor, 350 V; Oct 1RF Vpp, 750 V; sheath gas (N_2_) temperature, 325 °C, sheath gas flow, 11 L/min; nozzle voltage, 2000 V; and capillary voltage, 4000 V. The acquisition mode was Auto MS/MS (Seg), and the scan mass range was *m/z* 250–1500 for both MS^1^ and MS^2^. Acquisition rate/time were 3 spectra/s and 333.3 ms/spectrum for MS^1^, and 4 spectra/s and 250 ms/spectrum for MS^2^ acquisition, respectively. The collision energy was set by applying the “use formula” method: Collision energy = (slope) * (*m/z*)/100 + offset, and the slope was 6.67 and the offset was − 10. Top 3 precursors showing an absolute intensity not less than 500 counts and the relative threshold 10% (to the intensity of the base peak ion) were automatedly selected for the MS/MS fragmentation. The obtained MS data were analyzed by using the MassHunter Workstation Qualitative Analysis software (version 10.0, Agilent).

#### Three DDA-MS^2^ approaches for performance comparison in characterizing the ginsenosides in PJM

The performance of three DDA approaches (**M1** to **M3**) in acquiring the collision-induced dissociation (CID)-MS^2^ data of PJM was compared. The general settings in Auto MS/MS (**M1**) were common for all three methods, and their only difference was the absence or inclusion of different PILs. In M1, no PIL was input, and intensity ranking-based selection of top 3 most intense precursors was achievable. M2 and M3 could be regarded as the PIL-including improved DDA strategies. For M2, the target masses (including 305 different *m/z* values, in total) corresponding to the known 579 ginsenosides, by considering the different adduct forms (e.g. both [M−H]^−^ and [M−H + HCOOH]^−^ for the neutral ginsenosides; [M−H]^−^ for the acidic saponins such as the OA-type and malonylated), were input. For M3, the target masses (103 different *m/z* values; Additional file 1: Table S2) resulting from the screening of the high-accuracy MS^1^ data of PJM with MDF were input.

Development of the “Ginsenoside Sieve” was generally consistent with our previous report [44], which was based on the fixed variation range MDF and the in-house ginsenoside library. In detail, these 579 ginsenosides collected in the in-house database were in accordance with 185 different masses after removing the repeated values. The integer mass and decimal mass were distinguished by using the *mod* and *trunc* functions of Excel. The variation range, {Decimal mass − 10 mDa, Decimal mass + 10 mDa}, combined with the integer mass, could generate a sieve for ginsenosides. The established “Ginsenoside Sieve” was utilized to screen target *m/z* values from the MS^1^ raw data of PJM processed by the MassHunter Workstation software.

### Mass spectrometry imaging conditions

Fresh raw PJM sample was briefly rinsed with water and the fibrous roots on the surface were removed. Then, 2.5% Carboxymethyl cellulose (CMC), as the embedding medium, was dropped into the mold until the PJM slice sample was completely covered. Tin-foil mold was then immediately transferred to a refrigerator at − 80 °C for 20 min. The rhizome tissue was cryo-sectioned into the 40-µm thick slices at − 20 °C on a cryostat microtome (Leica CM3050 S, Leica Biosystems Nussloch GmbH, Germany), and further mounted onto an indium tin oxide (ITO) glass microscope slide. The PJM slice sample was brought to 4 °C refrigerator before the analysis.

A Xevo G2-XS QTOF mass spectrometer equipped with a DESI source (Waters Corporation) was used for MSI, operating in the negative mode in the mass range of *m/z* 100–1500. The spray solvent was 90% (v/v) methanol containing 0.1% FA delivered at a flow rate of 2 µL/min. A nebulizing gas pressure of 5 bar, cone voltage of 60 V, capillary voltage of − 4.5 kV, and ion source temperature of 130 °C, were set. The spatial resolution was set at 200 μm × 200 μm with a rate of 600 μm/s according to the sample dimensions. The imaging data were processed and viewed using the High Definition Imaging (HDI) software (Version 1.6, Waters Corporation, Manchester, UK).

## Results

### Development of a UHPLC/QTOF-MS approach for the systematic characterization of ginsenosides in PJM

Sufficient chromatographic separation is crucial to expose those minor components in an herbal extract, thus benefitting their characterization by LC–MS. The reversed-phase UHPLC conditions, such as the stationary phase, column temperature, and gradient elution program, were optimized to well resolve the multicomponents from PJM. We compared the selectivity of ten candidate C18-bonding columns from Waters and Agilent (detailed information in Additional file 1: Table S3; maintained at 30 °C) on the resolution of the PJM saponins. These columns have different silica gel cores and bonding techniques, and their performance was evaluated in terms of the resolution and selectivity. As illustrated in Additional file 1: Fig. S1, the tested columns displayed significantly different selectivity on the components of PJM, and comparatively, the CSH C18, BEH Shield RP18, and Zorbax SB-Aq columns, could better separate the major chromatographic peaks showing more balanced peak distribution. In addition, when evaluated by the amount of those resolvable peaks, the Zorbax Eclipse Plus C18 (462), Zorbax Extend C18 (441), and BEH Shield RP18 (431), were ranked among the top three. Taken together, we intended to select the BEH Shield RP18 column, in this work. On this basis, the influence of column temperature (including 30, 35, 40, and 45 °C) on the chromatographic separation of PJM components was evaluated in the next step (Additional file 1: Fig. S2). The higher temperature elevated the elution power of the mobile phase, and thereby could weaken the retention of PJM ginsenosides on the selected BEH Shield RP18 column. Under the same gradient elution program, the setting at 30 °C showed a much stronger retaining ability to the main peaks, and therefore, the column temperature was set at 30 °C for the further optimization steps.

Appropriate ion-source parameters can ensure high ion response of the target analytes, and suitable collision energy is able to dissociate more fragments from the precursors that are crucial to the structure elucidation by LC–MS. The ion-source parameters (involving nozzle voltage, capillary voltage, and Fragmentor) and the collision energy on the 6550 QTOF mass spectrometer, operating in the negative mode, were optimized by a series of single-factor experiments. Influences by variation of the nozzle voltage (500‒2000 V), capillary voltage (3.0‒4.0 kV), and Fragmentor (350‒400 V), were tested by comparing the average peak areas of 19 reference compounds through three parallel determinations. They included the mono-glycosides: 20(*R*)-ginsenoside Rh1, compound K, and pseudoginsenoside RT5 (24*R*); di-glycosides: ginsenosides Rg1, -Rg3, 24(*R*)-pseudoginsenoside F11, chikusetsusaponin IVa, and zingibroside R1; tri-glycosides: ginsenosides Re, -Rd, -Ro, notoginsenoside R1, malonylfloralginsenoside Rd5, pseudoginsenoside RT1, and chikusetsusaponin IV; tetra-glycosides: malonylginsenosides Rb1, -Rb2, and ginsenoside Rb3; penta-glycoside: ginsenoside Ra1. Generally, all the index compounds gave the increasing ion response with the elevating of nozzle voltage in the tested variation range, and accordingly, 2000 V of nozzle voltage was selected (Additional file 1: Fig. S3A). Capillary voltage for the 6550 QTOF mass spectrometer directly affects the ion transmission, and its optimization targets to improve the transmission efficiency and, simultaneously, to avoid the in-source fragmentation of the precursor ions. The effects of various capillary voltage values varying from 3.0 to 4.0 kV were assessed, and a similar variation trend in the ion response of the ginsenoside precursors was obtained when alternating the capillary voltage. Capillary voltage of 4.0 kV could enable the highest intensity for all the index compounds (Additional file 1: Fig. S3B), under which no severe in-source fragmentation was observed. Fragmentor is the voltage applied to the outlet of the capillary on the 6550 QTOF mass spectrometer, and in this experiment, six different fragmentor values (350/360/370/380/390/400 V) were tested to evaluate its variation on the ion response of ginsenosides. Evidently, the best response for 17 ginsenosides was enabled at the fragmentor of 350 V, except for ginsenoside Ra1 at 390 V and zingibroside R1 at 370 V. We thus selected the fragmentor at 350 V as the best condition (Additional file 1: Fig. S3C).

Collision energy is an important parameter affecting the quality of MS^2^ spectra and the number of components that can be characterized [45]. In the Auto MS/MS mode, three different settings can be optional to set collision energy (use fixed collision energy, use table, and use formula). Considering the structural diversity of ginsenosides contained in PJM, we chose the formula pattern to set collision energy, by which the dissociation degree difference among different masses of ginsenosides could be taken into account. In this section, the intensity ratios of the sapogenin ions to precursors (with 1‒5 sugars) were used as the indicator. A two-step optimization approach was utilized. The first step of optimization was to probe the appropriate collision energy (constant value) for the ginsenosides glycosylated with different number of sugars, and the setting of 30/40/45/50/55/60 V was evaluated. Balanced MS^2^ spectra containing the precursors and sapogenin ions could represent high-quality MS^2^ spectra beneficial to the structural elucidation. For the mono-glycosidic ginsenoside structures, such as 20(*S*)-ginsenoside F1 (*t*_R_ 17.59 min, *m/z* 637.4322), collision energy higher than 40 V could completely dissociate the deprotonated precursors ([M‒H]^‒^), and according to the ion intensity ratio, 30 V was determined as the best collision energy. In the similar manner, optimal collision energy for ginsenosides with different numbers of sugars was gained. In the next step, we used the “use formula” method (collision energy = (slope) × (*m*/*z*)/100 + offset) to set the collision energy according to the results of single-level collision energy examination. The components at the low mass end (600 *m/z*) corresponded to optimal collision energy of 30/40 V, while those at the high mass end (1200 *m/z*) were consistent with 55/60/65/70 V. In accordance, different slopes and offsets were obtained, generating total eight equations to characterize the collision energy. As shown in Additional file 1: Fig. S3D, a ratio of [Sapogenin−H]^‒^/[M−H]^‒^ between 0.5 and 1.5 could partially indicate the balanced MS^2^ spectrum, and therefore, formula 6 was applicable to the CID-MS/MS of the ginsenosides containing 1‒3 sugars, whereas formulae 7 and 8 were more suitable to the dissociation of tetra- and penta-glycosidic ginsenosides. By considering the MS^2^ spectra quality of all nine index ginsenosides and the ability to better dissociate the mono- to penta-glycosidic ginsenosides, formula 4 with a slope of 6.67 and an offset of − 10 was regarded as the best condition. Additional file 1: Fig. S4 shows the balanced MS/MS fragmentation induced by the optimized collision energy.

### Establishment and performance comparison for three MS/MS data acquisition approaches appliable to characterize the ginsenosides in PJM

The addition of PIL has been demonstrated as powerful to target more interesting components, thus greatly improving the coverage of the DDA strategy [25, 44]. Aside from the Auto MS/MS method (**M1**), another two PIL-DDA approaches were established. Based on our in-house ginsenoside database recording 579 known ginsenosides that are ever reported from various *Panax* species, the direct input of 305 *m/z* values as a table included in Auto MS/MS setting was considered as **M2**. In addition, integration of MDF and the in-house ginsenoside library could enable a sieve to filter the target *m/z* values from the full-scan MS^1^ data of the PJM extract, which were included in Auto MS/MS setting, as **M3** (detailed information of the precursors are provided in Additional file 1: Table S2). The established MDF tool, as exhibited in Additional file 1: Fig. S5A, could efficiently filter the targeted 103 *m/z* values (Additional file 1: Fig. S5B). Evidently, the filtered target masses only occupied a small proportion of the precursor ions detected from PJM. By the newly established **M2** and **M3**, those targeted precursor ions with weak intensity were endowed with the high priority to trigger the MS/MS fragmentation, causing the sensitive characterization of target components. Moreover, when no target masses were scanned, the instrument could automatedly select the unknown top 3 precursors for the MS/MS fragmentation, similar to the work pattern of “*if idle, pick others*” available on the Thermo Fisher Scientific Q-Exactive Q-Orbitrap mass spectrometer [46].

**Fig. 3:**
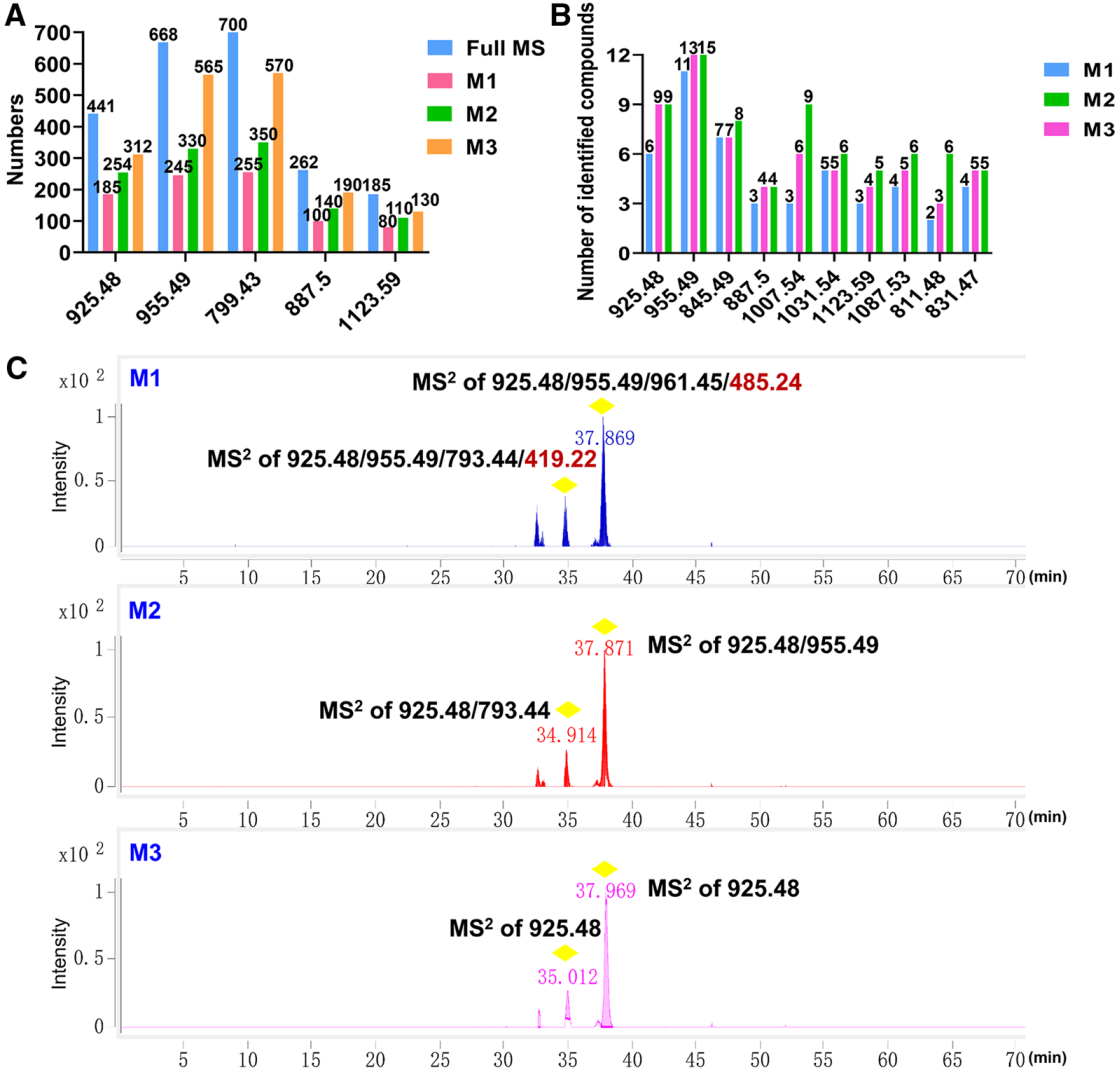
Comparing the performance of three DDA approaches in characterizing ginsenosides from PJM.

The present ginsenoside sieve-based PIL-DDA approach showed significant advantages in the targeted compounds characterization over the conventional DDA. Firstly, in the case of five characteristic saponin precursor ions (*m/z* 925.48, 955.49, 799.43, 887.50, and 1123.59), 70% or more (not dereplicated) of them could be selected for the MS/MS fragmentation by **M3**. However, this ratio was less than 50% by using the Auto MS/MS (**M1**), with **M2** ranked in the middle (Fig. 3A). From the ultimate characterization results, **M3** was also superior to the other two, as evidenced by the main components of PJM (using ten *m/z* values; Fig. 3B). The target mass at *m*/*z* 925.48 was taken as an example for illustration (Fig. 3C). The **M3** approach only captured the MS^2^ information of the target mass (*m/z* 925.48), while the **M1** and **M2** methods also covered the other precursor ions, even those non-saponin ions. These pieces of evidence could justify that the ginsenoside sieve-based PIL-DDA approach was the most effective in characterizing ginsenosides from PJM. We also found the complementarity of **M2** and **M3** in the characterization of PJM saponins, and thus their results were pooled to maximize the characterization results.

### Systematic characterization of ginsenosides from PJM by UHPLC/QTOF-MS

By searching the in-house database and comparing with 92 reference compounds and the related literature, we were able to identify or tentatively characterize 272 compounds from PJM (Additional file 1: Table S4). According to the structure differences on the sapogenins and the presence or absence of malonyl substitution, these ginsenosides could be classified into the PPD, PPT, OA, octillol-type (OT), malonylated (referring to those with the sapogenin other than OA/PPD/PPT), C-17 side-chain varied, and the others. Those compounds identified by comparison with the reference compounds are indicated in Additional file 1: Fig. S6.

#### OA-type

**Fig. 4:**
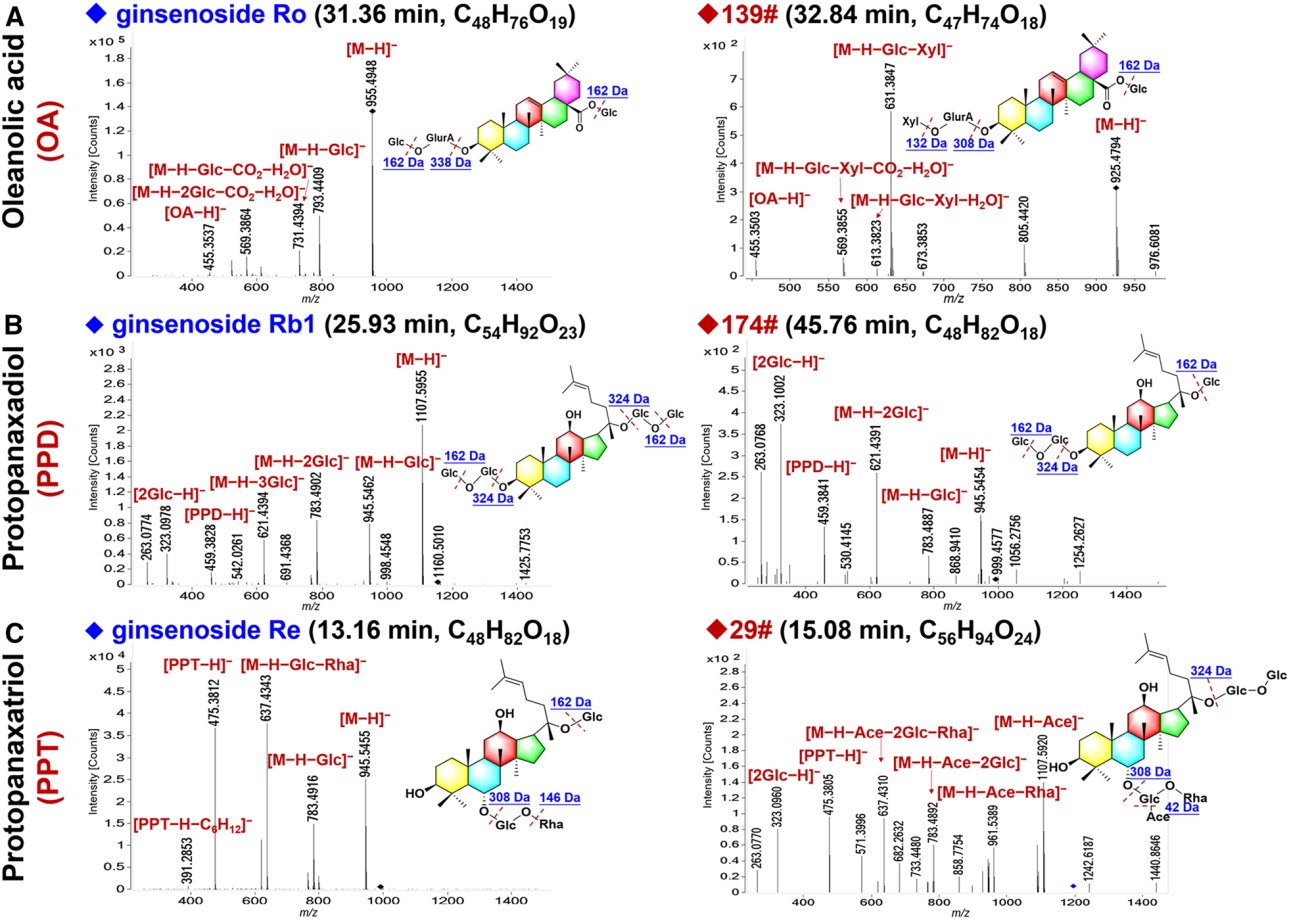
Structural elucidation of different subclasses of ginsenosides in PJM based on the negative CID-MS^2^ data

PJM contains abundant OA-type ginsenosides, and a total of 75 OA ginsenosides (27.57% of the total amount) were characterized. Different from the neutral ginsenosides [47, 48], the OA-type produced rich deprotonated precursors, but almost no formic acid (FA)-adducts. The 3-OH or/and 28-COOH are the common glycosylation sites for the OA saponins [1]. The diagnostic sapogenin ion at *m/z* 455.35 in the MS^2^ spectrum was one of the identification features for the OA-type ginsenosides. These MS features could be proven by a reference compound, ginsenoside Ro (corresponding to compound **135#**; *t*_R_ 31.36 min; C_48_H_76_O_19_). As shown in Fig. 4A, the abundant deprotonated precursor ion was observed at *m/z* 955.4948, and the fragments including *m/z* 793.4409, 731.4394, and 569.3864, were readily detected, which were assigned as [M−H−Glc]^−^, [M−H−Glc−CO_2_−H_2_O]^−^, and [M−H−2Glc−CO_2_−H_2_O]^−^, respectively. The fragment ion at *m/z* 455.3537 was consistent with the deprotonated OA sapogenin. In the case of an unknown compound **139**# (*t*_R_ 32.84 min), the deprotonated precursor was observed at *m/z* 925.4794, which could suggest the molecular formula as C_47_H_74_O_18_ (mass error: 3.78 ppm). Successive cleavages of Glc and Xyl (294 Da) on the precursor ion could generate the fragment at *m/z* 631.3847, while the fragments at *m/z* 613.3823 ([M−H−Glc−Xyl−H_2_O]^−^) and 569.3855 ([M−H−Glc−Xyl−CO_2_−H_2_O]^−^) were consistent with the product ions after the eliminating of H_2_O and CO_2_, respectively. Consequently, compound **139**# was tentatively characterized as an isomer of chikusetsusaponin IV, by searching the in-house database and comparing the retention behavior. In summary, the elution time of these OA-type ginsenosides ranged from 19.06 to 59.84 min, and the structures contained 1–4 sugars (e.g. Glc, Rha, Xyl, and GlurA). In addition, diverse acylation forms were found in the OA-type saponins characterized from PJM, such as the butenyl (But, 68.03 Da, **247#**), acetyl (Ace, 42.01 Da, **172#**/**180#**/**187#**/**192#**/**204#**/**211#**/**212#**/**235#**/**236#**), and malonyl (Mal, 86.00 Da, **158#**/**162#**/**171#**/**197#**/**210#**/**224#**/**265#**/**267#**).

#### PPD-type

We could characterize 22 PPD-type (8.09% of the total amount) ginsenosides from PJM. Unlike the OA type, neutral PPD ginsenosides could readily form the FA-adducts as the predominant precursors [47]. The fragmentation features of PPD ginsenosides could be evidenced by the reference compound, ginsenoside Rb1 (consistent with compound **114**# in Additional file 1: Table S4: *t*_R_ 25.93 min, C_54_H_92_O_23_) with the deprotonated precursor ion observed at *m/z* 1107.5955. The characteristic fragmentation by neutral loss of four Glc residues was observed yielding a sapogenin fragment at *m/z* 459.3828 (Fig. 4B), which was the diagnostic sapogenin ion for the PPD-type ginsenosides. In the case of an unknown saponin, compound **174**# (*t*_*R*_ 45.76 min) gave the deprotonated precursor ion at *m/z* 945.5454, which could indicate the molecular formula as C_48_H_82_O_18_ (1.48 ppm). Witnessed in the MS^2^ spectrum, the product ions at *m/z* 783.4887, 621.4391, and 459.3841, could indicate the attachment of three Glc residues to the PPD sapogenin. In combination with searching the in-house database and comparing with the literature, we tentatively characterized compound **174**# as chikusetsusaponin FK7 or its isomer (an isomer of Rd). The elution time range of the PPD-type ginsenosides was 17.19–47.85 min, and their structures containing 1–5 sugars (Glc, Rha, and Xyl). The acylation forms of acetyl (Ace, 42.01 Da, **48#**/**216#**/**217#**) and malonyl (Mal, 86.00 Da, **126#**/**147#**/**153#**/**161#**/**176#**/**181#**/**184#**/**191#**/**193#**/**196#**/**201#**) were also characterized for the PPD-type saponins in PJM.

#### PPT-type

The PPT-type ginsenosides, similar to the PPD type, could also form the FA-adducts as the predominant precursors. Moreover, the MS/MS fragmentation behavior observed for the PPT-type was quite similar to that of the PPD-type, but the difference was the yielded sapogenin ion at *m/z* 475.38. In this work, 52 PPT-type ginsenosides (19.12% of the total amount) got characterized. These fragmentation features could be embodied by a reference compound, ginsenoside Re (corresponding to **24**#; *t*_R_ 13.16 min; C_48_H_82_O_18_). As illustrated in Fig. 4C, it gave the rich deprotonated precursor ion at *m/z* 945.5455, which, upon CID-MS/MS, two major fragments at *m/z* 783.4916 and 637.4343 were observed, which were assigned as [M−H−Glc]^−^ and [M−H−Glc−Rha]^−^, respectively. In addition, its MS^2^ spectrum displayed the sapogenin ions at *m/z* 475.3812 and 391.2853 associated with the PPT sapogenin [47]. In the case of an unknown compound **29**# (*t*_R_ 15.08 min), it gave the FA-adduct precursor at *m*/*z* 1195.6150, suggesting the molecular formula as C_56_H_94_O_24_ (2.87 ppm). Its MS^2^ spectrum displayed the product ions at *m*/*z* 1107.5920, 961.5389, 783.4892, 637.4310, and 475.3805, which were assigned to [M−H−Ace]^−^, [M−H−Rha]^−^, [M−H−2Glc]^−^, [M−H−2Glc−Rha]^−^, and [PPT−H]^−^, respectively. By searching the in-house database, compound **29**# was tentatively characterized as yesanchinoside F or its isomer (PPT-3Glc-Rha-Ace). The elution time of the PPD-type ginsenosides was in the range of 3.96–50.87 min, and their structures contained 1–4 sugars (Glc, Rha, and Xyl). The acetyl (Ace, 42.01 Da, **29#**/**44#**/**54#**/**62#**/**159#**/**206#**) and malonyl (Mal, 86.00 Da, **207#**/**150#**/**157#**/**163#**/**39#**/**43#**/**53#**) were characterized in the PPT-type ginsenosides of PJM.

#### Others

Aside from these three subtypes depicted above, 16 OT-type (5.88% of the total), 19 malonylated (6.99%), and 35 C-17 side-chain varied ginsenosides (12.87%), and 53 others (19.49%), were characterized from PJM, with their information detailed in Additional file 1: Table S4.

### Spatial distribution of ginsenosides in the rhizome of PJM by DESI-MSI

**Fig. 5:**
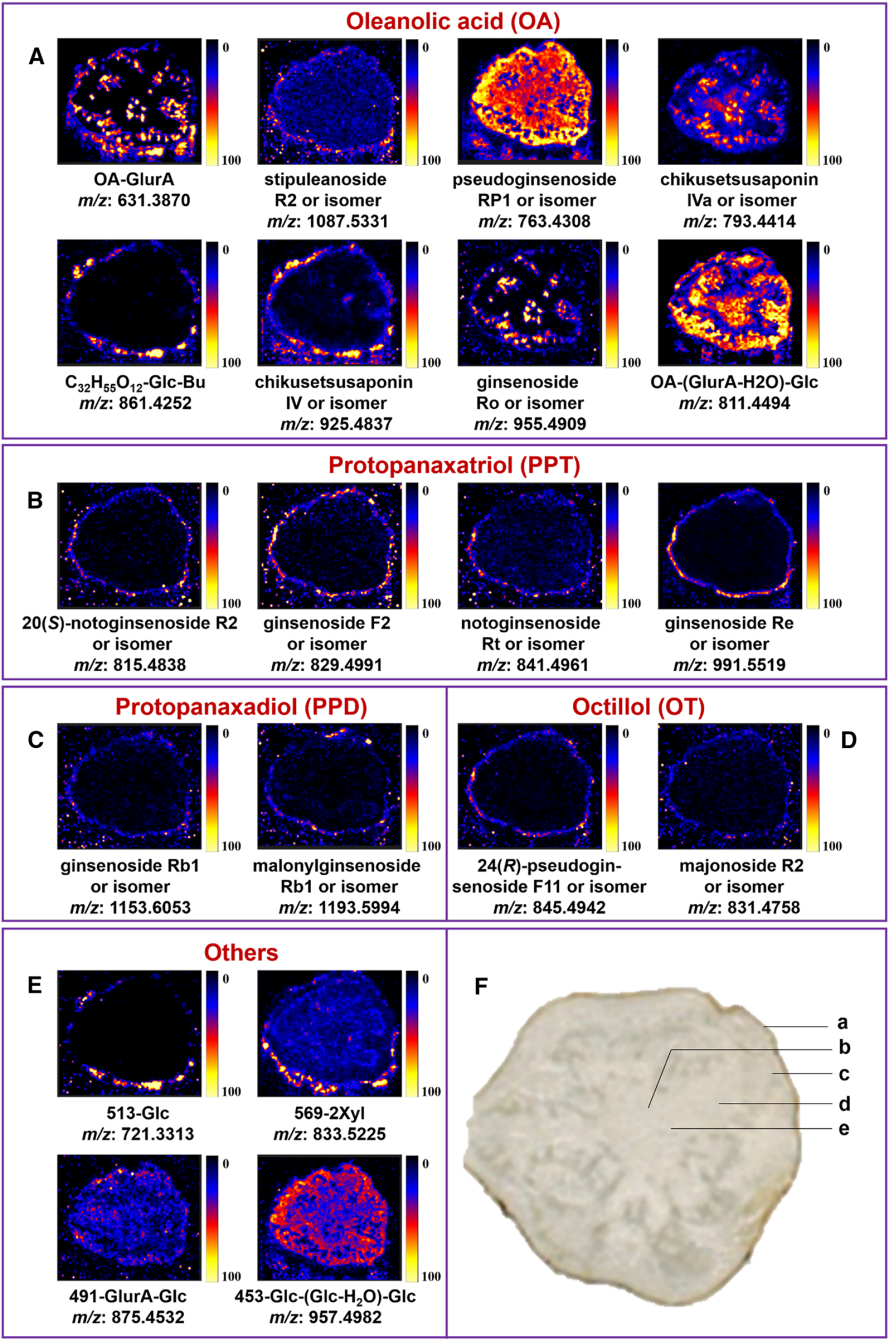
The spatial distribution of representative ginsenosides in the rhizome of PJM, including the OA-type (**A**), PPT-type (**B**), PPD-type (**C**), OT-type (**D**), and the others (**E**), and the optical image of the rhizome of PJM (**F**, a-cork; b-medulla; c-phloem; d-cambium; e-xylem)

The in situ spatial distribution of various ginsenosides from PJM was studied based on DESI-MSI with a mass range of *m/z* 100–1500 Da in the negative ion mode. The images in Fig. 5F show the main compartments of the rhizome of PJM, including the cork, phloem, cambium, xylem, and medulla (the boundary is not clear) [49]. Figure 5A–E illustrates the MS images of representative ginsenosides, including the OA-type, PPT-type, PPD-type, the OT-type, and the others. Furthermore, the most prevalent group of ginsenosides observed in PJM was the OA-type using DESI-MSI, which was in line with their relative high content characterized by UHPLC/QTOF-MS.

Variations in the distribution of OA-type saponins were readily discerned in the rhizome of PJM (Fig. 5A). As the most significant saponin occurring to PJM, chikusetsusaponin IVa (*m/z* 793.4414, [M−H]^−^)/isomer and ginsenoside Ro (*m/z* 955.4943, [M−H]^−^)/isomer displayed higher ion intensity in the cork and phloem regions, which suggested the biosynthesis of these compounds might occur in phloem and then transfer to the cork for the accumulation. Chikusetsusaponin IV (*m/z* 925.4837, [M−H]^−^)/isomer was highly concentrated in the cork. Stipuleanoside R2 (*m/z* 1087.5331, [M−H]^−^)/isomer and an unknown saponin C_32_H_55_O_12_-Glc-But (*m/z* 861.4252, [M−H]^−^) were distributed in the cork. In the cork and phloem, another two unknown OA-type ginsenosides, OA-GlurA (*m/z* 631.3870, [M−H]^−^) and OA-(GlurA-H_2_O)-Glc (*m/z* 811.4494, [M−H]^−^), were detected. The distribution of pseudoginsenoside RP1 (*m/z* 763.4308, [M−H]^−^)/isomer was detected in all the rhizome tissues.

Additionally, some main PPT ginsenosides were concentered in the cork tissue. The characteristic 20(*S*)-notoginsenoside R2 (*m/z* 815.4838, [M−H+HCOOH]^−^)/isomer, ginsenoside F2 (*m/z* 829.4991, [M−H+HCOOH]^−^)/isomer, notoginsenoside Rt (*m/z* 841.4961, [M−H]^−^)/isomer, and ginsenoside Re (*m/z* 991.5519, [M−H+HCOOH]^−^)/isomer, could be readily observed (Fig. 5B). Furthermore, some PPD and OT ginsenosides were also visualized by DESI-MSI, which matched the distribution of the PPT ginsenosides in the cork. The distribution of ginsenoside Rb1 (*m/z* 1153.6053, [M−H+HCOOH]^−^)/isomer, malonylginsenoside Rb1 (*m/z* 1193.5994, [M−H]^−^)/isomer (Fig. 5C), 24(*R*)-pseudoginsenoside F11 (*m/z* 845.4942, [M−H]^−^)/isomer, and majonoside R2 (*m/z* 831.4758, [M−H]^−^)/isomer was observed in the cork (Fig. 5D). Moreover, the distribution of four other ginsenosides (*m/z* 721.3313, [M−H+HCOOH]^−^; *m/z* 833.5225, [M−H]^−^; *m/z* 875.4532, [M−H+HCOOH]^−^; *m/z* 957.4982, [M−H]^−^) was varied in the rhizome tissues, mainly in the cork and phloem (Fig. 5E).

## Discussion

As the chemical components in the herbal extracts are very complex, the systematic characterization of herbal components represents a great challenge in analytical chemistry. Compared with the other popular ginseng species, such as *P. ginseng* and *P. notoginseng* [21, 24, 25, 44, 47, 48], studies on the composition of ginsenosides in PJM, particularly aimed to discover more unknown saponins, are very rare. As shown in Additional file 1: Fig. S5, the majority of the ionized molecules in PJM are out of our targets. In this context, by LC–MS using the conventional DDA strategy (such as Auto MS/MS, **M1**), only a small proportion of the target precursor ions can trigger the MS/MS fragmentation, yielding the fragments information for the structural characterization. Many useless acquisitions may occur, targeting those nontarget precursors or tautologically recording the fragmentation of the diverse adducts, the multiply charged ion species, or even the in-source fragmentation ions [44]. Therefore, to improve the coverage on the ginsenosides of interest, aside from the commonly used Auto MS/MS (**M1**), we also developed two PIL-including DDA methods (**M2** and **M3**). The final results could testify the great potency of the established PIL-DDA approaches (235 ones characterized by **M2** and 226 by **M3**, compared with 152 based on **M1**).

It is noted that, some other strategies, such as the multi-dimensional chromatography (MDC) and DIA, have been demonstrated as powerful in the in-depth characterization of herbal components [19–21, 45, 50]. MDC integrates more dimensions of separations, which are mostly orthogonal, can greatly enhance the peak capacity and the selectivity of separation. However, the operations may become more complicated (such as the off-line MDC) or specific instruments are needed (for the on-line mode MDC) restricting its accessibility [20, 25, 51]. DIA is an MS^2^ acquisition technique, which, in theory, can acquire the MS/MS fragmentation information for all the ionized precursors [45, 50]. However, a step of deconvolution to match between the precursors and the fragments is necessary, and the quality of the obtained MS^2^ spectra is not as good as those obtained by DDA [52]. In contrast, by including PIL in DDA, the coverage can be greatly improved with high-quality MS^2^ spectra obtained, and the involved operations are not as complicated as those in MDC.

MSI can depict the spatial distribution of the bioactive components in the medicinal herbs. By DESI-MSI analysis, in the current work, the location of the identified ginsenosides in the rhizome of PJM was unveiled, which were primarily concentrated in the cork and phloem tissues. The cork is the outermost protective tissue of a plant stem, while the phloem is the tissue responsible for transporting sugars and other organic nutrients throughout the plant [53]. It is thus possible that some ginsenosides play important roles in the defense mechanism or nutrient transport within the PJM plant, leading to the higher accumulation of ginsenosides in the cork and phloem regions [54]. The underlying mechanism requires more research in the future.

The UHPLC/QTOF-MS approach is powerful in the rapid characterization of ginsenosides, while DESI-MSI gives detailed tissue distribution information for the characterized ginsenosides in the rhizome of PJM. Integration of these two analytical techniques thus provides more reliable characterization results, including the structure and the location in tissue. However, we’re also aware of the possible limitations encountered in our work, such as the restricted reliability in ginsenosides identification with isomers difficult to be differentiated, the resolution of DESI-MSI, and the sensitivity in imaging ginsenosides. More validation experiments will be designed and conducted to solve these issues.

## Conclusion

By integrating UHPLC/QTOF-MS and DESI-MSI, the compositions of ginsenosides in the rhizome of PJM were comprehensively elucidated and their spatial distribution in the rhizome slice was depicted. The inclusion of PIL in DDA could largely improve the coverage on ginsenosides of interest, compared with the conventional DDA strategy. Moreover, the PIL resulting from different methodologies (direct input of the known ginsenoside information and the use of a ginsenoside sieve) could embody the complementarity in characterizing the ginsenosides from PJM in such a complex chemical matrix. A total of 272 ginsenosides were identified or tentatively characterized from PJM, and 138 thereof were considered to be unknown for the *Panax* genus. DESI-MSI experiments indicated the differentiated distribution of different subtypes of saponins, but mainly concentrated in the cork layer and phloem. This study provides insight into the elaborate composition of ginsenosides and their spatial distribution in the rhizome of PJM, which is thus beneficial to the quality control and its further development.

### Supplementary Information

**Additional file 1: Table S1.** Information for 92 ginsenoside reference compounds used in this work. **Table S2.** Detailed information of the precursor ions screened by the ginsenoside sieve from the high-accuracy MS^1^ data of PJM. **Table S3.** Information of ten commercial chromatographic columns as the candidates for selecting the stationary phase in establishing the UHPLC/QTOF-MS approach. **Table S4.** Information for the 272 ginsenosides characterized from PJM. **Figure S1.** Selection of the stationary phase for the reversed-phase UHPLC separation of the multicomponents from PJM. **Figure S2.** Development of the UHPLC/QTOF-MS system: comparison of the column temperature using the BEH Shield RP18 column. **Figure S3.** Optimization of three key ion-source parameters and the collision energy on the 6550 QTOF mass spectrometer operating in the negative mode for acquiring the CID-MS^2^ data of PJM saponins. **Figure S4.** The MS^1^ and MS^2^ spectra of six ginsenosides involving one to five sugars, showing the balanced MS/MS fragmentation by the optimized collision energy. **Figure S5.** Establishment of a ginsenoside sieve by mass defect filtering and the in-house ginsenoside library and its application to PJM to screen the precursors of ginsenosides with target *m/z* values. **Figure S6.** Base peak intensity (BPI) chromatograms of PJM in the negative ESI mode.

## Data Availability

The original contributions presented in the study are included in the article/Additional file, further inquiries can be directed to the corresponding authors.

## Abbreviations

PJM: *Panax japonicus* var. *major*
UHPLC/QTOF-MS: Ultra-high performance liquid chromatography/quadrupole time-of-flight mass spectrometry
DESI-MSI: Desorption electrospray ionization-mass spectrometry imaging
PIL: Precursor ions list
DDA: Data-dependent acquisition
OA: Oleanolic acid
PPD: Protopanaxadiol
PPT: Protopanaxatriol
OT: Octillol
LC–MS: Liquid chromatography/mass spectrometry
MSI: Mass spectrometry imaging
MALDI: Matrix-assisted laser desorption ionization
DESI: Desorption electrospray ionization
MDF: Mass defect filtering
ITO: Indium tin oxide
HDI: High definition imaging
