# Supplementary material for: Characterization of ginsenosides from Panax japonicus var. major (Zhu-Zi-Shen) based on ultra-high performance liquid chromatography/quadrupole time-of-flight mass spectrometry and desorption electrospray ionization-mass spectrometry imaging
Source: Chin Med. 2023 Sep 8;18:115. doi: 10.1186/s13020-023-00830-9 (PMC10486018; doi:10.1186/s13020-023-00830-9)
Supplement: Supplementary file 1 — Additional file 1: Table S1. Information for 92 ginsenoside reference compounds used in this work. Table S2. Detailed information of the precursor ions screened by the ginsenoside sieve from the high-accuracy MS1 data of PJM. Table S3. Information of ten commercial chromatographic columns as the candidates for selecting the stationary phase in establishing the UHPLC/QTOF-MS approach. Table S4. Information for the 272 ginsenosides characterized from PJM. Figure S1. Selection of the stationary phase for the reversed-phase UHPLC separation of the multicomponents from PJM. Figure S2. Development of the UHPLC/QTOF-MS system: comparison of the column temperature using the BEH Shield RP18 column. Figure S3. Optimization of three key ion-source parameters and the collision energy on the 6550 QTOF mass spectrometer operating in the negative mode for acquiring the CID-MS2 data of PJM saponins. Figure S4. The MS1 and MS2 spectra of six ginsenosides involving one to five sugars, showing the balanced MS/MS fragmentation by the optimized collision energy. Figure S5. Establishment of a ginsenoside sieve by mass defect filtering and the in-house ginsenoside library and its application to PJM to screen the precursors of ginsenosides with target m/z values. Figure S6. Base peak intensity (BPI) chromatograms of PJM in the negative ESI mode. [file 13020_2023_830_MOESM1_ESM.pdf]

<*Chinese Medicine*>

Additional file 1

Characterization of ginsenosides from *Panax japonicus* var. *major* (Zhu-Zi-Shen) based on ultra-high performance liquid chromatography/quadrupole time-of-flight mass spectrometry and desorption electrospray ionization-mass spectrometry imaging

Meiting Jiang [1,2,†], Xiaohang Li [1,2,†], Yuying Zhao [1,2,†], Yadan Zou [1,2], Maoli Bai [1,2], Zhiming Yang [3], Wei Wang [1,2], Xiaoyan Xu [1,2], Hongda Wang [1,2], Wenzhi Yang [1,2,3,*], Qinhua Chen [3,*], Dean Guo [1,2,3,4]

[1] National Key Laboratory of Chinese Medicine Modernization, State Key Laboratory of Component-based Chinese Medicine, Tianjin University of Traditional Chinese Medicine, 10 Poyanghu Road, Tianjin 301617, China

[2] Haihe Laboratory of Modern Chinese Medicine, Tianjin University of Traditional Chinese Medicine, 10 Poyanghu Road, Tianjin 301617, China

[3] Shenzhen Baoan Authentic TCM Therapy Hospital, Shenzhen 518101, China

[4] Shanghai Research Center for Modernization of Traditional Chinese Medicine, National Engineering Laboratory for TCM Standardization Technology, Shanghai Institute of Materia Medica, Chinese Academy of Sciences, 501 Haike Road, Shanghai 201203, China

[†] Meiting Jiang, Xiaohang Li and Yuying Zhao contributed equally to this work.

[*] Correspondence: wzyang0504@tjutcm.edu.cn; cqh77@163.com.

Contents

Figure S1 Selection of the stationary phase for the reversed-phase UHPLC separation of the multicomponents from PJM. **A**-Total ion current chromatograms obtained on ten candidate sub-2 μm particles packed columns; **B**-scatter plot of the resolved peaks with their numbers annotated (obtained by processing the high-resolution MS^1 data of PJM by MassHunter).

Figure S2 Development of the UHPLC/QTOF-MS system: comparison of the column temperature using the BEH Shield RP18 column.

Figure S3 Optimization of three key ion-source parameters (**A**-nozzle voltage; **B**-capillary voltage; **C**-fragmentor) and the collision energy (**D**) on the 6550 QTOF mass spectrometer operating in the negative mode for acquiring the CID-MS^2 data of PJM saponins.

Figure S4 The MS^1 and MS^2 spectra of six ginsenosides involving one to five sugars, showing the balanced MS/MS fragmentation by the optimized collision energy.

Figure S5 Establishment of a ginsenoside sieve (**A**) by mass defect filtering and the in-house ginsenoside library and its application to PJM to screen the precursors of ginsenosides with target *m/z* values (**B**).

Figure S6 Base peak intensity (BPI) chromatograms of PJM in the negative ESI mode. The peaks characterized with the aid of reference compounds are annotated in red.

14: 20-*O*-glucoginsenoside Rf; **17**: notoginsenoside R1; **22**: ginsenoside Rg1; **24**: ginsenoside Re; **64**: vinaginsenoside R8; **68**: vinaginsenoside R4 ; **80**: 24(*R*)-pseudoginsenoside F11; **83**: ginsenoside Rf; **91**: notoginsenoside R4; **92**: 20(*S*)-notoginsenoside R2; **102**: 20(*S*)-ginsenoside Rh1; **103**: 20(*S*)-ginsenoside Rg2; **106**: ginsenoside F3; **109**: 20(*R*)-ginsenoside Rg2; **114**: ginsenoside Rb1; **124**: ginsenoside Rc; **126**: malonylginsenoside Rb1; **135**: ginsenoside Ro; **141**: ginsenoside Rb3; **151**: chikusetsusaponin IV; **153**: malonylginsenoside Rb2; **155**: pseudoginsenoside RT1; **161**: malonylfloralginsenoside Rc1; **164**: ginsenoside Rd; **168**: chikusetsusaponin IVa; **176**: malonylfloralginsenoside Rd5; **182**: gypenoside XVII; **199**: notoginsenoside Fd; **202**: ginsenoside F4; **214**: ginsenoside F2; **221**: zingibroside R1.

Table S1 Information for 92 ginsenoside reference compounds used in this work.

Table S2 Detailed information of the precursor ions screened by the ginsenoside sieve from the high-accuracy MS^1 data of PJM.

Table S3 Information for the 272 ginsenosides characterized from PJM.

Table S4 Information of ten commercial chromatographic columns as the candidates for selecting the stationary phase in establishing the UHPLC/QTOF-MS approach.

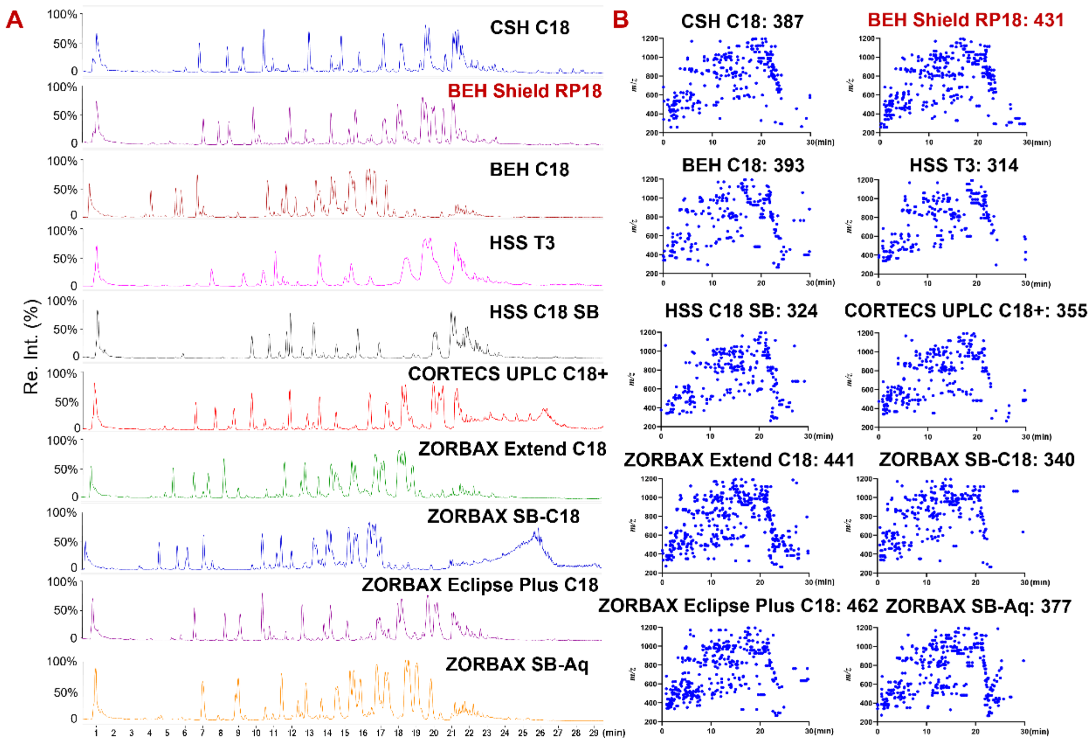

Figure S1 Selection of the stationary phase for the reversed-phase UHPLC separation of the multicomponents from PJM. **A**-Total ion current chromatograms obtained on ten candidate sub-2 µm particles packed columns; **B**-scatter plot of the resolved peaks with their numbers annotated (obtained by processing the high-resolution MS^1 data of PJM by MassHunter).

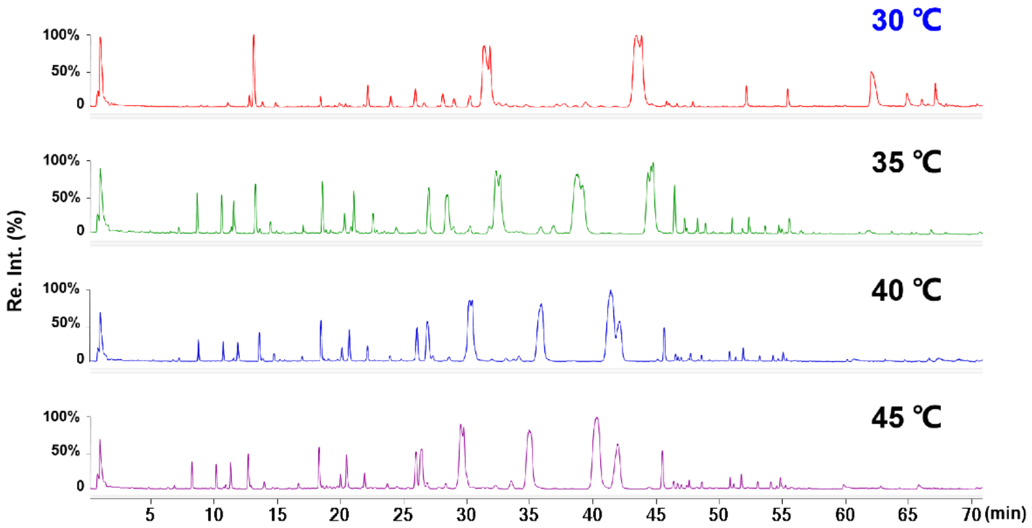

Figure S2 Development of the UHPLC/QTOF-MS system: comparison of the column temperature using the BEH Shield RP18 column.

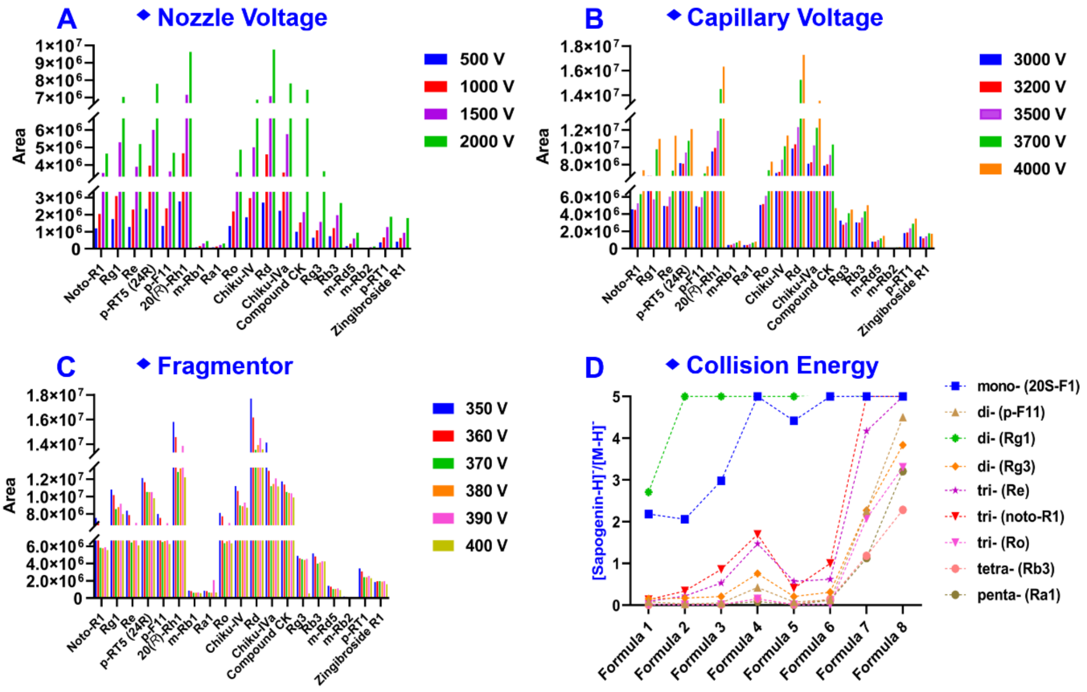

Figure S3 Optimization of three key ion-source parameters (**A**-nozzle voltage; **B**-capillary voltage; **C**-fragmentor) and the collision energy (**D**) on the 6550 QTOF mass spectrometer operating in the negative mode for acquiring the CID-MS^2 data of PJM saponins.

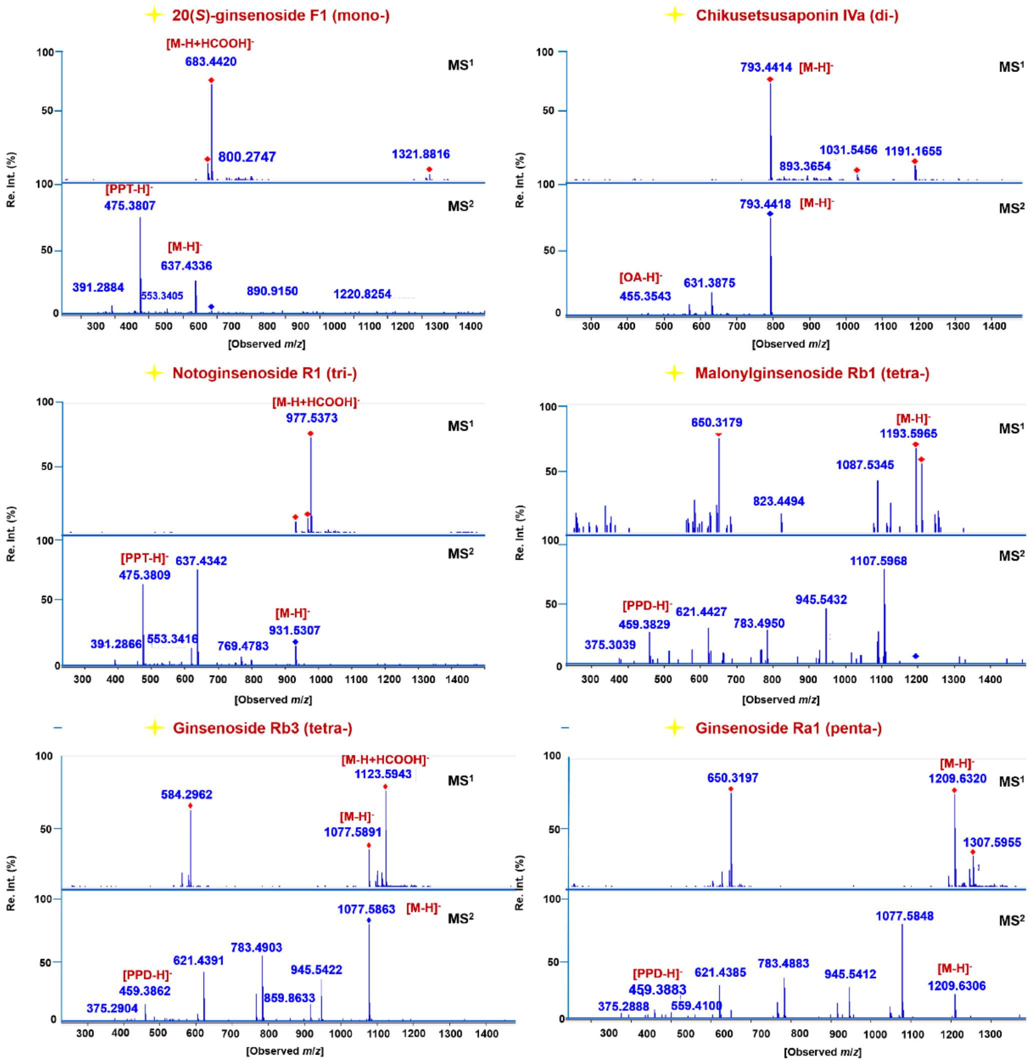

Figure S4 The MS^1 and MS^2 spectra of six ginsenosides involving one to five carbohydrate moieties, showing the balanced MS/MS fragmentation by the optimized collision energy.

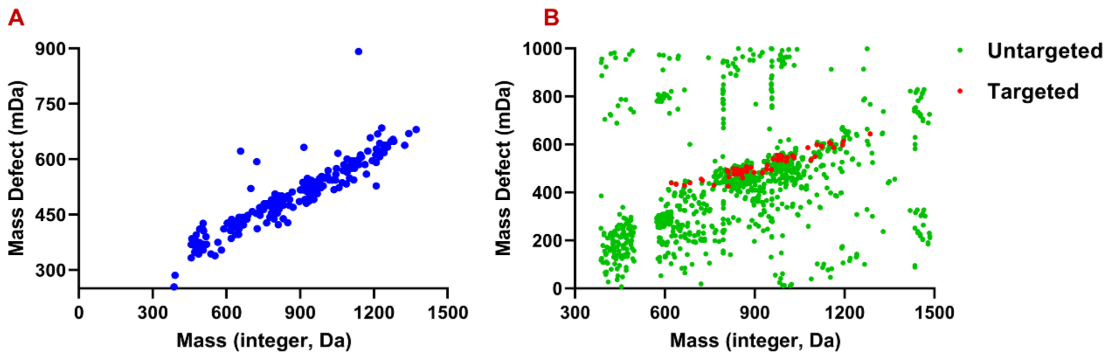

Figure S5 Establishment of a ginsenoside sieve (**A**) by mass defect filtering and the in-house ginsenoside library and its application to PJM to screen the precursors of ginsenosides with target *m/z* values (**B**).

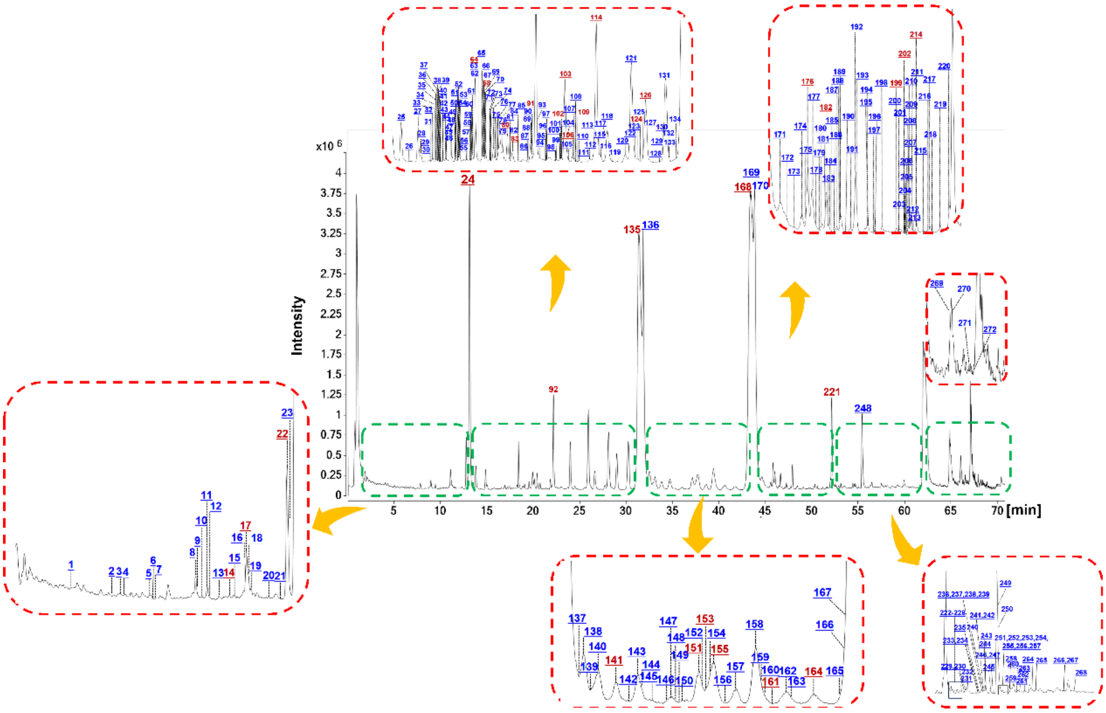

Figure S6 Base peak intensity (BPI) chromatograms of PJM in the negative ESI mode. The peaks characterized with the aid of reference compounds are annotated in red.

14: 20-*O*-glucoginsenoside Rf; **17**: notoginsenoside R1; **22**: ginsenoside Rg1; **24**: ginsenoside Re; **64**: vinaginsenoside R8; **68**: vinaginsenoside R4 ; **80**: 24(*R*)-pseudoginsenoside F11; **83**: ginsenoside Rf; **91**: notoginsenoside R4; **92**: 20(*S*)-notoginsenoside R2; **102**: 20(*S*)-ginsenoside Rh1; **103**: 20(*S*)-ginsenoside Rg2; **106**: ginsenoside F3; **109**: 20(*R*)-ginsenoside Rg2; **114**: ginsenoside Rb1; **124**: ginsenoside Rc; **126**: malonylginsenoside Rb1; **135**: ginsenoside Ro; **141**: ginsenoside Rb3; **151**: chikusetsusaponin IV; **153**: malonylginsenoside Rb2; **155**: pseudoginsenoside RT1; **161**: malonylfloralginsenoside Rc1; **164**: ginsenoside Rd; **168**: chikusetsusaponin IVa; **176**: malonylfloralginsenoside Rd5; **182**: gypenoside XVII; **199**: notoginsenoside Fd; **202**: ginsenoside F4; **214**: ginsenoside F2; **221**: zingibroside R1.

Table S1 Information for 92 ginsenoside reference compounds used in this work.

No.	Trivial Name	M.F.	Exact Mass	Subtype	Lot Number	No.	Trivial Name	M.F.	Exact Mass	Subtype	Lot Number
1	20(*S*)-protopanaxatriol	$C_{30}H_{52}O_4$	476.3866	PPT	STA-10042005	47	notoginsenoside S	$C_{63}H_{106}O_{30}$	1342.6769	PPD	DST210201-138
2	20(*R*)-ginsenoside Rh1	$C_{36}H_{62}O_9$	638.4394	PPT	DST201029-018	48	notoginsenoside Fh1	$C_{63}H_{106}O_{30}$	1342.6769	PPD	/
3	20(*S*)-ginsenoside Rh1	$C_{36}H_{62}O_9$	638.4394	PPT	DST190703-005	49	notoginsenoside D	$C_{64}H_{108}O_{31}$	1372.6875	PPD	/
4	ginsenoside F3	$C_{41}H_{70}O_{13}$	770.4816	PPT	DST170712-027	50	notoginsenoside T	$C_{64}H_{108}O_{31}$	1372.6875	PPD	/
5	20(*S*)-notoginsenoside R2	$C_{41}H_{70}O_{13}$	770.4816	PPT	DST190301-053	51	malonylfloralginsenoside Rb3	$C_{56}H_{92}O_{25}$	1164.5928	PPD	/
6	ginsenoside F5	$C_{41}H_{70}O_{13}$	770.4816	PPT	DST200803-029	52	malonylfloralginsenoside Rc1	$C_{56}H_{92}O_{25}$	1164.5928	PPD	/
7	20(*R*)-notoginsenoside R2	$C_{41}H_{70}O_{13}$	770.4816	PPT	DST200815-052	53	(20*S*), (24*R*)-Ocotillol	$C_{30}H_{52}O_5$	492.3815	OT	DST190608-025
8	20(*S*)-ginsenoside Rg2	$C_{42}H_{72}O_{13}$	784.4973	PPT	DST190517-010	54	pseudoginsenoside RT5	$C_{36}H_{62}O_{10}$	654.4343	OT	3184
9	20(*R*)-ginsenoside Rg2	$C_{42}H_{72}O_{13}$	784.4973	PPT	/	55	pseudoginsenoside F11	$C_{42}H_{72}O_{14}$	800.4922	OT	DST200228-008
10	ginsenoside Rf	$C_{42}H_{72}O_{14}$	800.4922	PPT	ST02520120MG	56	zingibroside R1	$C_{42}H_{66}O_{14}$	794.4453	OA	G210Y0613B1
11	ginsenoside Rg1	$C_{42}H_{72}O_{14}$	800.4922	PPT	8797	57	chikusetsusaponin IVa	$C_{42}H_{66}O_{14}$	794.4453	OA	1396
12	chikusetsusaponin L5	$C_{46}H_{78}O_{17}$	902.5239	PPT	/	58	pseudoginsenoside RT1	$C_{47}H_{74}O_{18}$	926.4875	OA	PS000491
13	notoginsenoside R1	$C_{47}H_{80}O_{18}$	932.5345	PPT	7571	59	chikusetsusaponin IV	$C_{47}H_{74}O_{18}$	926.4875	OA	DST210203-035
14	ginsenoside Re	$C_{48}H_{82}O_{18}$	946.5501	PPT	/	60	ginsenoside Ro	$C_{48}H_{76}O_{19}$	956.4981	OA	5695
15	vinaginsenoside R4	$C_{48}H_{82}O_{19}$	962.545	PPT	DST200915-068	61	panaxadiol	$C_{30}H_{52}O_3$	460.3916	Others	DST190109-037
16	20-*O*-glucoginsenoside-Rf	$C_{48}H_{82}O_{19}$	962.545	PPT	GT21113007	62	panaxatriol	$C_{30}H_{52}O_4$	476.3866	Others	DST181120-038
17	20(*R*)-protopanaxadiol	$C_{30}H_{52}O_3$	460.3916	PPT	RFS-Y12102008021	63	ginsenoside Rh3	$C_{36}H_{60}O_7$	604.4339	Others	DST190701-012
18	20(*S*)-protopanaxadiol	$C_{30}H_{52}O_3$	460.3916	PPT	66-21-53200	64	ginsenoside Rk2	$C_{36}H_{60}O_7$	604.4339	Others	DST190525-35
19	20(*S*)-ginsenoside Rh2	$C_{36}H_{62}O_8$	622.4445	PPT	ST00980120MG	65	ginsenoside Rk3	$C_{36}H_{60}O_8$	620.4288	Others	DST2008013-036
20	20(*R*)-ginsenoside Rh2	$C_{36}H_{62}O_8$	622.4445	PPT	DSTDR001901	66	ginsenoside Rh4	$C_{36}H_{60}O_8$	620.4288	Others	DST210801-020
21	compound K	$C_{36}H_{62}O_8$	622.4445	PPT	R-028-161216	67	pseudoginsenoside Rh2	$C_{36}H_{62}O_8$	622.4445	Others	DST190708-009

No.	Trivial Name	M.F.	Exact Mass	Subtype	Lot Number	No.	Trivial Name	M.F.	Exact Mass	Subtype	Lot Number
22	20(*S*)-ginsenoside Rg3	$C_{42}H_{72}O_{13}$	784.4973	PPT	RFS-R01011804026	68	ginsenoside Rh7	$C_{36}H_{60}O_{9}$	636.4237	Others	DST190510-021
23	ginsenoside F2	$C_{42}H_{72}O_{13}$	784.4973	PPT	4147	69	ginsenoside Rh8	$C_{36}H_{60}O_{9}$	636.4237	Others	DST190708-022
24	ginsenoside Rd2	$C_{47}H_{80}O_{17}$	916.5396	PPD	DST180613-016	70	notoginsenoside T5	$C_{41}H_{68}O_{12}$	752.4711	Others	G210Y0917B1
25	notoginsenoside Fd	$C_{47}H_{80}O_{17}$	916.5396	PPD	DST190708-136	71	ginsenoside F4	$C_{42}H_{70}O_{12}$	766.4867	Others	DST180627-028
26	20(*R*)-notoginsenoside Ft1	$C_{47}H_{80}O_{17}$	916.5396	PPD	DST190516-051	72	ginsenoside Rk1	$C_{42}H_{70}O_{12}$	766.4867	Others	RFS-R05402011013
27	notoginsenoside Fe	$C_{47}H_{80}O_{17}$	916.5396	PPD	DST201025-037	73	ginsenoside Rg5	$C_{42}H_{70}O_{12}$	766.4867	Others	DST200321-032
28	vinaginsenoside R18	$C_{47}H_{80}O_{17}$	916.5396	PPD	/	74	ginsenoside Rg6	$C_{42}H_{70}O_{12}$	766.4867	Others	DST190416-033
29	ginsenoside Rd	$C_{48}H_{82}O_{18}$	946.5501	PPD	ST05970120	75	gypenoside A	$C_{46}H_{74}O_{17}$	898.4926	Others	DST210317-013
30	gypenoside XVII	$C_{48}H_{82}O_{18}$	946.5501	PPD	7669	76	quinquenoside L3	$C_{47}H_{80}O_{18}$	932.5345	Others	/
31	malonylfloralginsenoside Rd5	$C_{51}H_{84}O_{21}$	1032.5505	PPD	/	77	notoginsenoside NL-B1	$C_{47}H_{80}O_{18}$	932.5345	Others	/
32	ginsenoside Rc	$C_{53}H_{90}O_{22}$	1078.5924	PPD	ST04640120	78	notoginsenoside NL-G1	$C_{47}H_{80}O_{18}$	932.5345	Others	/
33	vinaginsenoside R7	$C_{53}H_{90}O_{22}$	1078.5924	PPD	/	79	notoginsenoside NL-H1	$C_{47}H_{80}O_{18}$	932.5345	Others	/
34	ginsenoside Rb2	$C_{53}H_{90}O_{22}$	1078.5924	PPD	ST02490120	80	notoginsenoside NL-H2	$C_{47}H_{80}O_{18}$	932.5345	Others	/
35	ginsenoside Rb3	$C_{53}H_{90}O_{22}$	1078.5924	PPD	DST200315-008	81	5,6-didehydroginsenoside Rd	$C_{48}H_{80}O_{18}$	944.5345	Others	DST190702-010
36	ginsenoside Rb1	$C_{54}H_{92}O_{23}$	1108.6029	PPD	DSTDR000601	82	notoginsenoside NL-A1	$C_{47}H_{80}O_{19}$	948.5294	Others	/
37	malonylginsenoside Rb2	$C_{56}H_{92}O_{25}$	1164.5928	PPD	/	83	vinaginsenoside R8	$C_{48}H_{82}O_{19}$	962.545	Others	DST190401-059
38	malonylginsenoside Rc	$C_{56}H_{92}O_{25}$	1164.5928	PPD	/	84	gypenoside XLVI	$C_{48}H_{82}O_{19}$	962.545	Others	DST201026-111
39	malonylginsenoside Rb1	$C_{57}H_{94}O_{26}$	1194.6033	PPD	/	85	gypenoside XLIX	$C_{52}H_{86}O_{21}$	1046.5662	Others	RFS-J00411-809028
40	notoginsenoside Fc	$C_{58}H_{98}O_{26}$	1210.6346	PPD	1908109	86	notoginsenoside NL-C3	$C_{53}H_{88}O_{23}$	1092.5716	Others	/
41	notoginsenoside FP2	$C_{58}H_{98}O_{26}$	1210.6346	PPD	/	87	floranotoginsenoside A	$C_{53}H_{90}O_{23}$	1094.5873	Others	/
42	ginsenoside Ra1	$C_{58}H_{98}O_{26}$	1210.6346	PPD	DST200726-039	88	a	$C_{53}H_{90}O_{23}$	1094.5873	Others	/

No.	Trivial Name	M.F.	Exact Mass	Subtype	Lot Number	No.	Trivial Name	M.F.	Exact Mass	Subtype	Lot Number
43	ginsenoside Ra2	$C_{58}H_{98}O_{26}$	1210.6346	PPD	DST200613-040	89	b	$C_{53}H_{90}O_{23}$	1094.5873	Others	/
44	notoginsenoside R4	$C_{59}H_{100}O_{27}$	1240.6452	PPD	NS-ZB0271-DT05	90	notoginsenoside NL-B3	$C_{58}H_{98}O_{27}$	1226.6295	Others	/
45	notoginsenoside Fa	$C_{59}H_{100}O_{27}$	1240.6452	PPD	6042	91	notoginsenoside NL-G2	$C_{58}H_{98}O_{27}$	1226.6295	Others	/
46	ginsenoside Ra3	$C_{59}H_{100}O_{27}$	1240.6452	PPD	DST190108-041	92	notoginsenoside NL-H3	$C_{58}H_{98}O_{27}$	1226.6295	Others	/

a: 3-*O*-*β*-D-glucopyranosyl-(1→2)-*β*-D-glucopyranosyl-20-*O*-*β*-D-xylopyranosyl (1→6)-*β*-D-glucopyranosyl-darmmar-25-ene-3*β*,12*β*,20*S*,24*S*-tetraol

b: 3-*O*-*β*-D-glucopyranosyl-(1→2)-*β*-D-glucopyranosyl-20-*O*-*β*-D-xylopyranosyl (1→6)-*β*-D-glucopyranosyl-darmmar-25-ene-3*β*,12*β*,20*S*,24*R*-tetrao

Table S2 Detailed information of the precursor ions screened by the ginsenoside sieve from the high-accuracy MS^1 data of PJM.

RT (min)	Precursors	RT (min)	Precursors	RT (min)	Precursors	RT (min)	Precursors
9.01	1007.5454	18.27	861.4879	28.12	955.4955	39.66	1163.5874
9.47	1153.6036	18.43	1077.5863	28.48	887.5031	40.67	855.4773
9.97	831.4769	18.93	887.5030	28.99	1149.6077	43.89	811.4252
10.14	831.4764	18.94	1007.5450	30.24	841.4983	45.72	991.5494
10.40	1007.5457	19.11	841.4964	30.79	1087.5346	45.82	987.5551
10.96	977.5346	19.28	1007.5443	31.34	955.4941	45.82	1031.5471
11.01	845.4932	19.56	887.5044	31.85	955.4925	46.16	1163.5866
11.10	977.5368	19.57	873.4869	32.10	1123.5900	46.16	925.4818
11.19	861.4887	19.60	831.4767	32.54	1087.5358	46.76	1031.5444
11.30	1123.5929	19.67	843.4767	32.94	925.4818	47.27	725.4494
11.42	1123.5927	19.86	989.5354	33.11	955.4940	47.27	637.4340
12.01	977.5339	19.94	831.4787	33.78	925.4810	47.85	1031.5459
13.34	831.4764	19.94	875.5025	33.79	1123.5927	47.85	987.5553
13.83	875.5021	20.76	845.4936	34.72	859.4605	48.34	975.5553
13.83	831.4787	21.36	845.4928	34.76	925.4827	48.34	939.4972
14.88	845.4943	22.03	1285.6449	35.18	955.4930	48.48	1031.5445
15.03	831.4773	23.70	843.4769	35.91	941.5134	48.75	961.5399
15.07	1195.6134	23.87	683.4396	35.99	1193.5983	49.72	721.4555
16.00	1025.5547	24.00	897.4841	36.60	855.4767	50.59	829.4987
16.57	1031.5448	25.59	857.4924	37.11	925.4839	51.87	811.4866
16.93	961.5388	25.80	955.4942	37.32	1163.5869	52.66	811.4874
16.99	1005.5294	25.92	1107.5992	37.55	955.4933	53.44	763.4298
17.20	843.4762	26.20	1101.5499	37.72	925.4840	53.53	807.4918
17.39	845.4926	26.59	1087.5348	38.60	855.4780	56.82	665.4290
17.46	1031.5451	26.65	955.4942	38.60	811.4866	68.74	621.4399
17.91	813.4655	27.74	1087.5357	39.28	871.5073		

Table S3 Information of the 272 ginsenosides characterized from PJM.

No.	RT (min)	Observed *m/z*	ppm	Formula	Adducts	MS^2	Identification	Type	M1	M2	M3
1	3.96	1007.5430	−0.21	$C_{48}H_{82}O_{19}$	[M−H+HCOOH]$^-$	637.4367	notoginsenoside R3 or isomer (PPT-3Glc)	PPT		√	√
2	5.52	1023.5347	−3.47	$C_{48}H_{82}O_{20}$	[M−H+HCOOH]$^-$	797.4664,765.4303,651.4088,595.3612,577.6585	floralginsenoside J or isomer ($C_{36}H_{59}O_{10}$-Rha-H_2O-Glc)	C-17 side-chain varied		√	
3	5.98	993.5259	−1.79	$C_{47}H_{80}O_{19}$	[M−H+HCOOH]$^-$	947.4953,785.4668,653.4225,491.3769	vinaginsenoside R6 (24S) or isomer (OT-Glc-Xyl-Glc)	OT		√	
4	6.05	863.5007	−0.37	$C_{42}H_{74}O_{15}$	[M−H+HCOOH]$^-$	671.4401,509.3866,489.7366,415.7047,389.7561	quinquenoside L9 or isomer ($C_{30}H_{53}O_6$-Glc-Rha)	C-17 side-chain varied		√	
5[b]	7.10	849.4880	−6.62	$C_{41}H_{72}O_{15}$	[M−H+HCOOH]$^-$	803.4798,671.4406,509.3801	$C_{30}H_{53}O_6$-Glc-Xyl	others	√	√	√
6	7.19	1007.5431	−0.10	$C_{48}H_{82}O_{19}$	[M−H+HCOOH]$^-$	961.5497,653.4305	ginsenoside Re7 or isomer ($C_{36}H_{61}O_{10}$-Glc-Rha)	C-17 side-chain varied			√
7	7.30	863.5007	−1.19	$C_{42}H_{74}O_{15}$	[M−H+HCOOH]$^-$	817.4905,671.4372,509.3840	quinquenoside L9 or isomer ($C_{30}H_{53}O_6$-Glc-Rha)	C-17 side-chain varied	√	√	√
8	8.94	861.4863	1.22	$C_{42}H_{72}O_{15}$	[M−H+HCOOH]$^-$	617.1488,501.7517,471.6551,387.4013	ginsenoside Rg12 or isomer	C-17 side-chain varied		√	√
9	9.06	1007.5430	−0.21	$C_{48}H_{82}O_{19}$	[M−H+HCOOH]$^-$	961.5376,637.4367,475.3732	notoginsenoside R6 or isomer (PPT-3Glc)	PPT		√	√
10	9.23	831.4758	1.27	$C_{41}H_{70}O_{14}$	[M−H+HCOOH]$^-$	785.4716,653.4220,553.3374,491.3723	majonoside R2 or isomer (OT-Glc-Xyl)	OT		√	√
11	9.45	1153.6053	3.79	$C_{54}H_{92}O_{23}$	[M−H+HCOOH]$^-$	1107.5959,945.5455,783.4932,667.1900,637.4350,475.3808	ginsenoside Re8 or isomer (PPT-3Glc-Rha)	PPT	√	√	√

No.	RT (min)	Observed *m/z*	ppm	Formula	Adducts	MS^2	Identification	Type	M1	M2	M3
12	9.60	1007.5431	−0.10	$C_{48}H_{82}O_{19}$	[M−H+HCOOH]$^-$	781.4781,499.2889	vinaginsenoside R9 or isomer ($C_{42}H_{69}O_{13}$-H_2O-Glc)	C-17 side-chain varied			√
13	9.98	831.4783	4.45	$C_{41}H_{70}O_{14}$	[M−H+HCOOH]$^-$	785.4709,713.5431,653.4302,491.3747	majonoside R2 or isomer (OT-Glc-Xyl)	OT	√	√	√
14[a]	10.39	1007.5463	3.22	$C_{48}H_{82}O_{19}$	[M−H+HCOOH]$^-$	961.5372,799.4888,637.4321,475.3804	20-*O*-glucoginsenoside Rf	PPT	√	√	√
15	10.60	1123.5934	2.60	$C_{53}H_{90}O_{22}$	[M−H+HCOOH]$^-$	809.3475, 729.4182, 637.4317, 619.4252, 475.3810	floralginsenoside M or isomer (PPT-Rha-2Glc-Xyl)	PPT	√	√	√
16	10.99	845.4942	4.75	$C_{42}H_{72}O_{14}$	[M−H+HCOOH]$^-$	799.4839,653.4320,491.3749	24(*S*)-pseudoginsenoside F11 or isomer (OT-Glc-Rha)	OT	√	√	√
17[a]	11.12	977.5361	3.65	$C_{47}H_{80}O_{18}$	[M−H+HCOOH]$^-$	931.5250,799.4793,637.4349,475.3832	notoginsenoside R1	PPT	√	√	√
18	11.15	861.4884	−6.52	$C_{42}H_{72}O_{15}$	[M−H+HCOOH]$^-$	815.4795,653.4292,491.3749	24(*R*)-majoroside R1 or isomer (OT-2Glc)	OT	√	√	√
19	11.27	1123.5934	2.60	$C_{53}H_{90}O_{22}$	[M−H+HCOOH]$^-$	1077.5835, 987.4340, 945.5492, 931.5259, 921.3283, 689.2919, 637.4302, 619.4220, 517.3845, 475.3816	floralginsenoside M or isomer (PPT-Rha-2Glc-Xyl)	PPT	√	√	√
20	12.05	977.5361	3.65	$C_{47}H_{80}O_{18}$	[M−H+HCOOH]$^-$	637.4273,619.4255, 475.3841	ginsenoside Re4 or isomer (PPT-2Glc-Xyl)	PPT	√	√	√
21	12.53	1167.5794	−0.89	$C_{54}H_{90}O_{24}$	[M−H+HCOOH]$^-$	1121.5701,959.5263,797.4685,475.3654	notoginsenoside-B or isomer (PPT-GlurA-Rha-2Glc)	PPT		√	
22[a]	12.80	845.4942	4.75	$C_{42}H_{72}O_{14}$	[M−H+HCOOH]$^-$	799.4788, 637.4354,475.3815	ginsenoside Rg1	PPT	√	√	√
23[b]	12.85	897.4540	5.30	$C_{44}H_{68}O_{16}$	[M−H+HCOOH]$^-$	851.4011,689.2927,527.6242	527-Glc-Glc	others		√	
24[a]	13.16	991.5519	3.80	$C_{48}H_{82}O_{18}$	[M−H+HCOOH]$^-$	945.5455,783.4916,637.4343,475.3812,391.2853	ginsenoside Re	PPT	√	√	√

No.	RT (min)	Observed *m/z*	ppm	Formula	Adducts	MS²	Identification	Type	M1	M2	M3
25	13.82	831.4783	4.45	$C_{41}H_{70}O_{14}$	[M−H+HCOOH]⁻	785.4742,653.4305,491.3754	majonoside R2 or isomer (OT-Glc-Xyl)	OT	√	√	√
26	14.33	991.5497	1.48	$C_{48}H_{82}O_{18}$	[M−H+HCOOH]⁻	783.4902,637.4280	ginsenoside Rg18 or isomer ($C_{36}H_{61}O_9$-Rha-Glc)	PPT		√	√
27	14.86	845.4942	4.75	$C_{42}H_{72}O_{14}$	[M−H+HCOOH]⁻	799.4873,653.4314,491.3752	24(*R*)-pseudoginsenoside F11 isomer (OT-Glc-Rha)	OT	√	√	√
28	15.05	831.4783	4.45	$C_{41}H_{70}O_{14}$	[M−H+HCOOH]⁻	785.4745,653.4251,491.3671	majonoside R2 or isomer (OT-Glc-Xyl)	OT	√	√	√
29	15.08	1195.6150	2.87	$C_{56}H_{94}O_{24}$	[M−H+HCOOH]⁻	1107.5920,961.5389,783.4892,637.4310,475.3805,323.0960	yesanchinoside F or isomer (PPT-3Glc-Rha-Ace)	PPT	√	√	√
30	15.62	961.5385	0.76	$C_{47}H_{80}O_{17}$	[M−H+HCOOH]⁻	871.4200,607.0174,553.5532,475.3725	ginsenoside Rh24 or isomer (PPT-Xyl-Rha-Glc)	PPT		√	√
31	15.63	861.4863	1.22	$C_{42}H_{72}O_{15}$	[M−H+HCOOH]⁻	815.4853,635.4218,587.5241,491.3781	majonoside R1 or isomer (OT-2Glc)	OT		√	√
32	15.78	1137.5704	0.55	$C_{53}H_{88}O_{23}$	[M−H+HCOOH]⁻	797.4794,473.3687	notoginsenosides NL-C3 or isomer (473-3Glc-Xyl)	C-17 side-chain varied		√	
33	15.81	1005.5271	−0.52	$C_{48}H_{80}O_{19}$	[M−H+HCOOH]⁻	959.5075,779.4494,617.3982	vinaginsenoside R20 or isomer (617-2Glc-H_2O)	C-17 side-chain varied			√

No.	RT (min)	Observed *m/z*	ppm	Formula	Adducts	MS²	Identification	Type	M1	M2	M3
34	15.88	1169.5938	−2.05	$C_{54}H_{92}O_{24}$	$[M-H+HCOOH]^-$	1123.6062,799.4881,727.3385,475.3805	6−*O*−[*β*−D−glucopyranosyl−(1→2)−*β*−D−glucopyranosyl]−20−O−[*β*−D−glucopyranosyl−(1→4)−*β*−D−glucopyranosyl]−20(*S*)−protopanaxatriol or isomer (PPT−4Glc)	PPT		√	
35[b]	15.88	697.3072	−0.71	$C_{34}H_{50}O_{15}$	$[M-H]^-$	529.5935	529−Xyl−$2H_2O$	others	√	√	√
36	15.95	1123.5903	−2.05	$C_{54}H_{92}O_{24}$	$[M-H]^-$	799.4800,475.3881	6−*O*−[*β*−D−glucopyranosyl−(1→2)−*β*−D−glucopyranosyl]−20−*O*−[*β*−D−glucopyranosyl−(1→4)−*β*−D−glucopyranosyl]−20(*S*)−protopanaxatriol or isomer (PPT−4Glc)	PPT			√
37	16.03	991.5497	1.48	$C_{48}H_{82}O_{18}$	$[M-H+HCOOH]^-$	953.1910,815,4754,605.6578,475.3730	chikusetsusaponin FK1 or isomer (PPT−Glc−Rha−Glc)	PPT		√	
38	16.07	1025.5531	−0.71	$C_{48}H_{84}O_{20}$	$[M-H+HCOOH]^-$	979.5437,799.4855,637.4293,475.3730	ginsengenin−S3 or isomer (PPT−3Glc−H_2O)	PPT		√	√
39[b]	16.09	885.4890	3.56	$C_{45}H_{74}O_{17}$	$[M-H]^-$	637.4375,475.3810, 365.0285	PPT−2Glc−Mal	PPT	√		
40	16.20	887.5010	0.00	$C_{44}H_{74}O_{15}$	$[M-H+HCOOH]^-$	679.2731,621.5071,559.2789	notoginsenoside Rt or isomer (679−Glc)	PPT		√	√
41	16.27	989.5333	0.64	$C_{48}H_{80}O_{18}$	$[M-H+HCOOH]^-$	943.5333,781.4788,619.4260	ginsenoside Rh18 or isomer ($C_{36}H_{60}O_8$−2Glc)	C-17 side-chain varied		√	√

No.	RT (min)	Observed *m/z*	ppm	Formula	Adducts	MS^2	Identification	Type	M1	M2	M3
42	16.46	1139.5840	−1.37	$C_{53}H_{90}O_{23}$	$[M-H+HCOOH]^-$	799.4843,475.3855	chikusetsusaponin LM5 or isomer (PPT−3Glc−Xyl)	PPT		√	
43	16.56	1031.5462	2.91	$C_{51}H_{84}O_{21}$	$[M-H]^-$	945.5385,783.4784,619.4227,475.3822	malonylfloralginsenoside Rd2 or isomer (PPT−2Glc−Xyl−Mal)	PPT	√	√	√
44	16.61	987.5539	0.51	$C_{50}H_{84}O_{19}$	$[M-H]^-$	945.5446,637.4346,475.3779	pseudoginsenoside Rs1 or isomer (PPT−2Glc−Rha−Ace)	PPT		√	
45[b]	16.95	973.5039	2.06	$C_{48}H_{78}O_{20}$	$[M-H]^-$	811.4529, 793.4409, 769.4683, 749.4519, 633.3920, 631.3922,587.3975, 473.3645	631−2Glc−H_2O	others	√		
46	16.96	961.5402	2.62	$C_{47}H_{80}O_{17}$	$[M-H+HCOOH]^-$	753.4916,691.6643,607.4190,475.3806	ginsenoside Rh24 or isomer (PPT−Xyl−Rha−Glc)	PPT	√	√	√
47	17.00	1005.5303	2.81	$C_{48}H_{80}O_{19}$	$[M-H+HCOOH]^-$	959.5247,797.4770,635.4212,473.3642	vinaginsenoside R20 or isomer ($C_{30}H_{49}O_4$−3Glc）	C-17 side-chain varied	√	√	√
48	17.19	1165.6018	0.62	$C_{55}H_{92}O_{23}$	$[M-H+HCOOH]^-$	783.4972	ginsenoside Rs2 or isomer (783−Glc−Xyl−Ace)	PPD		√	
49	17.20	843.4780	4.06	$C_{42}H_{70}O_{14}$	$[M-H+HCOOH]^-$	797.4695,767.4572,635.4218,605.4015,443.3519	ginsenoside SL3 or isomer ($C_{36}H_{60}O_9$−Glc)	C-17 side-chain varied	√	√	√
50[b]	17.33	901.4789	−2.07	$C_{45}H_{74}O_{18}$	$[M-H]^-$	857.4964,653.4044,601.3413	$C_{39}H_{57}O_8$−Glc−Mal	Mal		√	
51	17.37	845.4942	4.75	$C_{42}H_{72}O_{14}$	$[M-H+HCOOH]^-$	799.4887, 669.1737, 637.4328, 571.3992, 551.9609, 427.3615, 383.7602	majoroside F2 or isomer ($C_{36}H_{61}O_9$−Glc)	C-17 side-chain varied	√	√	√
52	17.44	815.4809	1.29	$C_{42}H_{72}O_{15}$	$[M-H]^-$	635.0296	ginsenoside Rg12 or isomer (635−H_2O−Glc)	C-17 side-chain varied		√	

No.	RT (min)	Observed *m/z*	ppm	Formula	Adducts	MS²	Identification	Type	M1	M2	M3
53	17.46	1031.5462	2.91	$C_{51}H_{84}O_{21}$	$[M−H]^−$	945.5441,783.4940,637.4347,475.3815	malonylfloralginsenoside Rd3 or isomer (PPT−2Glc−Xyl−Mal)	PPT	√	√	√
54	17.47	1033.5623	3.44	$C_{50}H_{84}O_{19}$	$[M−H+HCOOH]^−$	921.3705, 785.4902, 679.4445, 545.3886, 475.3845, 391.2865	pseudoginsenoside Rs1 or isomer (PPT−2Glc−Rha−Ace)	PPT	√	√	√
55	17.88	961.5402	2.62	$C_{47}H_{80}O_{17}$	$[M−H+HCOOH]^−$	783.4961, 637.4324, 553.3323, 475.3805, 391.2865	ginsenoside Rh24 or isomer (PPT−Xyl−Rha−Glc)	PPT	√	√	√
56	17.92	815.4809	1.43	$C_{41}H_{70}O_{13}$	$[M−H+HCOOH]^−$	639.4280,475.3816	pseudoginsenoside RT3 or isomer (PPT−Xyl−Glc)	PPT		√	
57	17.95	813.4649	0.86	$C_{42}H_{70}O_{15}$	$[M−H]^−$	637.4279,475.3771	vinaginsenoside R25 or isomer (PPT−Glc−GlurA)	C-17 side-chain varied	√	√	√
58	18.11	1005.5286	1.04	$C_{48}H_{80}O_{19}$	$[M−H+HCOOH]^−$	959.5156,795.1950,517.6663,435.6859	vinaginsenoside R20 or isomer (517−Xyl−2Rha−H_2O)	C-17 side-chain varied		√	√
59	18.17	845.4907	0.37	$C_{42}H_{72}O_{14}$	$[M−H+HCOOH]^−$	799.4829,637.5156	notoginsenoside ST−8 or isomers (637−Glc)	C-17 side-chain varied			√
60	18.19	1007.5430	−0.21	$C_{48}H_{82}O_{19}$	$[M−H+HCOOH]^−$	961.5174,871.2662,661.5696,605.5724,517.1687	majoroside F1 or isomer (605−GlurA−H_2O−Glc)	C-17 side-chain varied		√	√
61	18.28	861.4884	3.80	$C_{42}H_{72}O_{15}$	$[M−H+HCOOH]^−$	815.4850,653.4350,491.3820	24(*R*)−majoroside R1 or isomer (OT−2Glc)	OT	√	√	√
62	18.43	1033.5623	3.44	$C_{50}H_{84}O_{19}$	$[M−H+HCOOH]^−$	987.5562,945.5451,783.4914,637.4352,475.3821	6'''−*O*−acetylginsenoside Re or isomer (PPT−2Glc−Rha−Ace)	PPT	√	√	√
63[b]	18.56	971.4890	2.84	$C_{48}H_{76}O_{20}$	$[M−H]^−$	809.4341, 791.4249, 629.3695, 611.3624, 603.3862, 585.3872, 541.3897, 521.3631,471.3507	$C_{30}H_{47}O_4$−GlurA−2Glc	others	√	√	√

No.	RT (min)	Observed *m/z*	ppm	Formula	Adducts	MS[2]	Identification	Type	M1	M2	M3
64[a]	18.65	1007.5431	−0.10	$C_{48}H_{82}O_{19}$	[M−H+HCOOH]⁻	781.4767,619.4192	vinaginsenoside R8	C-17 side-chain varied			√
65[b]	18.79	1047.5393	0.60	$C_{51}H_{84}O_{22}$	[M−H]⁻	651.4879,587.4061,475.3805	PPT−GlurA−3Xyl	PPT		√	
66[b]	18.89	959.5187	3.54	$C_{48}H_{80}O_{19}$	[M−H]⁻	783.4682	vinaginsenoside R20 or isomer (783−GlurA)	C-17 side-chain varied		√	
67	18.92	887.5041	3.71	$C_{44}H_{74}O_{15}$	[M−H+HCOOH]⁻	841.4923,799.4821,653.4234,547.0686,491.36 72	vinaginsenoside R1 or isomer (OT−Glc−Rha−Ace)	OT	√	√	√
68[a]	18.94	1007.5463	3.22	$C_{48}H_{82}O_{19}$	[M−H+HCOOH]⁻	961.5376,799.4888,637.4311,475.3848	vinaginsenoside R4	PPT	√	√	√
69[b]	19.06	811.4517	3.24	$C_{42}H_{68}O_{15}$	[M−H]⁻	811.4504, 649.3980, 631.3847, 605.4095, 587. 3971, 545.3948, 527.3737, 473.3688, 377.5151 , 359.3519	OA−H_2O−GlurA−Glc	OA	√	√	√
70	19.06	977.5332	0.54	$C_{47}H_{80}O_{18}$	[M−H+HCOOH]⁻	693.1063,541.1799,475.3855	notoginsenoside FP1 or isomer (PPT−2Glc−Xyl)	PPT		√	
71	19.26	1007.5463	3.22	$C_{48}H_{82}O_{19}$	[M−H+HCOOH]⁻	961.5389,781.4678,679.6255,475.3881	vinaginsenoside R4 isomer (PPT−3Glc)	PPT	√	√	√
72[b]	19.35	809.4358	2.94	$C_{42}H_{66}O_{15}$	[M−H]⁻	809.4362, 647.3824, 585.3792, 571.3638, 471. 3462, 341.7527	$C_{30}H_{47}O_4$−GlurA−Glc	others		√	√
73[b]	19.39	825.4315	3.83	$C_{42}H_{66}O_{16}$	[M−H]⁻	825.4301, 705.3880, 663.3729, 645.3637, 585. 3396, 469.1592	469−GlurA−Glc−H2O	others	√	√	√
74	19.54	887.5041	3.68	$C_{44}H_{74}O_{15}$	[M−H+HCOOH]⁻	841.4964,799.4898,653.4301,587.4001,491.37 49	vinaginsenoside R1 or isomer (OT−Glc−Rha−Ace)	OT	√	√	√
75	19.67	843.4780	4.06	$C_{42}H_{70}O_{14}$	[M−H+HCOOH]⁻	797.4679,635.421	ginsenoside SL3 or isomer ($C_{36}H_{60}O_9$−Glc)	C-17 side-chain varied	√	√	√

No.	RT (min)	Observed *m/z*	ppm	Formula	Adducts	MS²	Identification	Type	M1	M2	M3
76	19.79	975.5553	2.04	$C_{48}H_{82}O_{17}$	$[M-H+HCOOH]^-$	929.5478,783.4875,621.4327	vinaginsenoside R3 or isomer ($C_{36}H_{60}O_8$−Glc−Rha)	others		√	√
77	19.89	989.5363	3.84	$C_{48}H_{80}O_{18}$	$[M-H+HCOOH]^-$	943.5272,781.4698,619.4241,599.6814	ginsenoside Rh18 or isomer ($C_{36}H_{60}O_8$−2Glc)	C-17 side-chain varied	√	√	√
78	19.93	831.4783	4.45	$C_{41}H_{70}O_{14}$	$[M-H+HCOOH]^-$	785.4713,653.4293,491.3762	majonoside R2 or isomer (OT−Glc−Xyl)	OT	√	√	√
79[b]	20.10	987.4839	2.78	$C_{48}H_{76}O_{21}$	$[M-H]^-$	825.4306,807.4154,791.4215,451.3281	451−GlurA−2(Glc−H_2O)	others	√	√	√
80[a]	20.42	845.4942	4.75	$C_{42}H_{72}O_{14}$	$[M-H+HCOOH]^-$	799.4881,653.4293,491.3780	24(*R*)−pseudoginsenoside F11	OT	√	√	√
81[b]	20.62	811.4494	0.41	$C_{42}H_{68}O_{15}$	$[M-H]^-$	649.4250,455.3829	OA−H_2O−GlurA−Glc	OA		√	
82[b]	20.69	809.4358	2.94	$C_{42}H_{66}O_{15}$	$[M-H]^-$	809.4341, 689.3961, 647.3822, 629.3719, 587. 3810,471.3493	$C_{30}H_{47}O_4$−GlurA−Glc	others	√	√	√
83[a]	20.76	845.4942	4.75	$C_{42}H_{72}O_{14}$	$[M-H+HCOOH]^-$	799.4835,637.4365,475.3817	ginsenoside Rf	PPT	√	√	√
84[b]	21.15	809.4328	−0.76	$C_{42}H_{66}O_{15}$	$[M-H]^-$	647.3756,525.3586	$C_{36}H_{55}O_{10}$−Glc	others		√	
85[b]	21.16	721.3313	2.70	$C_{32}H_{52}O_{15}$	$[M-H+HCOOH]^-$	513.2747	513−Glc	others	√	√	√
86	21.29	845.4942	4.75	$C_{42}H_{72}O_{14}$	$[M-H+HCOOH]^-$	639.4366, 475.3811	ginsenoside Rf isomer (PPT−2Glc)	PPT	√	√	√
87[b]	21.69	813.4649	0.86	$C_{42}H_{70}O_{15}$	$[M-H]^-$	649.5933	vinaginsenoside R25 or isomer (649−H_2O−Rha)	C-17 side-chain varied		√	√
88	21.78	1117.5469	2.93	$C_{54}H_{86}O_{24}$	$[M-H]^-$	945.5430,793.4386,731.4378,621.4328	malonylfloralginsenoside Rd6 or isomer ($C_{36}H_{61}O_8$−2Glc−2Mal)	Mal	√	√	√
89[b]	21.98	841.4585	−0.77	$C_{42}H_{68}O_{14}$	$[M-H+HCOOH]^-$	795.4555,633.4013	$C_{36}H_{57}O_9$−Glc	others		√	√

No.	RT (min)	Observed *m/z*	ppm	Formula	Adducts	MS^2	Identification	Type	M1	M2	M3
90	22.00	1117.5446	0.89	$C_{54}H_{86}O_{24}$	[M−H]$^-$	945.5430,793.4385,731.4378,631.3926,621.4328	malonylfloralginsenoside Rd6 or isomers ($C_{36}H_{61}O_8$−2Glc−2Mal)	Mal	√	√	√
91[a]	22.02	1285.6466	2.58	$C_{59}H_{100}O_{27}$	[M−H+HCOOH]$^-$	1239.6480,1107.5953,1077.5734,783.4972,599.0188,459.3853	notoginsenoside R4	PPD	√	√	√
92[a]	22.19	815.4838	5.19	$C_{41}H_{70}O_{13}$	[M−H+HCOOH]$^-$	769.4772,637.4352,475.3823	20(*S*)−notoginsenoside R2	PPT	√	√	√
93	22.27	859.5048	−1.60	$C_{43}H_{74}O_{14}$	[M−H+HCOOH]$^-$	813.4240,637.4309,475.3794	notoginsenoside SY4 or isomer (PPT−Glc−GlurA)	C-17 side-chain varied			√
94[b]	22.48	807.4208	3.75	$C_{42}H_{64}O_{15}$	[M−H]$^-$	747.4318, 645.3651, 469.3355,	$C_{30}H_{45}O_4$−GlurA−Glc	others	√	√	√
95[b]	22.85	971.4890	3.84	$C_{48}H_{76}O_{20}$	[M−H]$^-$	971.4830, 809.4462, 747.4318, 629.3178, 479.3790	479−2Glc−Xyl−2H_2O	others	√	√	√
96	22.89	815.4809	1.43	$C_{41}H_{70}O_{13}$	[M−H+HCOOH]$^-$	637.4367,475.3817	chikusetsusaponin LM1 or isomer (PPT−Xyl−Glc)	PPT		√	√
97[b]	22.90	825.4315	3.83	$C_{42}H_{66}O_{16}$	[M−H]$^-$	825.4269, 777.4137, 645.3696	645−H_2O−Glc	others	√	√	√
98	23.43	665.3907	0.10	$C_{35}H_{56}O_9$	[M−H+HCOOH]$^-$	647.3912,485.6492	notoginsenoside ST6 or isomer	C-17 side-chain varied		√	√
99[b]	23.50	987.4811	−0.06	$C_{48}H_{76}O_{21}$	[M−H]$^-$	825.4270, 807.4232,573.2522, 529.2329	$C_{42}H_{63}O_{15}$−(Glc−H_2O)	others		√	√
100	23.69	1325.6419	2.71	$C_{62}H_{102}O_{30}$	[M−H]$^-$	1221.6215, 1191.6993, 1107.5979, 1079.5935, 997.8989, 945.5488, 765.4832, 621.4407, 559.3943, 459.3755	malonylnotoginsenoside R4 or isomer (PPD−4Glc−Xyl−Mal)	PPD	√	√	
101	23.72	843.4780	4.01	$C_{42}H_{70}O_{14}$	[M−H+HCOOH]$^-$	797.4680,635.4125,473.3687	ginsenoside SL3 or isomer (473−2Glc)	C-17 side-chain varied	√	√	√
102[a]	23.87	683.4363	−2.04	$C_{36}H_{62}O_9$	[M−H+HCOOH]$^-$	475.3854	20(*S*)−ginsenoside Rh1	PPT		√	√
103[a]	24.01	829.4991	4.59	$C_{42}H_{72}O_{13}$	[M−H+HCOOH]$^-$	783.4927,637.4361,475.3819	20(*S*)−ginsenoside Rg2	PPT	√	√	√

No.	RT (min)	Observed *m/z*	ppm	Formula	Adducts	MS^2	Identification	Type	M1	M2	M3
104[b]	24.09	873.5212	−1.18	$C_{45}H_{78}O_{16}$	$[M-H]^-$	727.2291	727−Rha	others			√
105	24.09	897.4816	−4.12	$C_{46}H_{74}O_{17}$	$[M-H]^-$	853.4570,631.3405	gypenoside A or isomer (631−H_2O−Glc−Mal)	Mal			√
106[a]	24.52	815.4809	1.43	$C_{41}H_{70}O_{13}$	$[M-H+HCOOH]^-$	637.3821,475.3805	ginsenoside F3	PPT		√	
107[b]	24.62	1017.5277	−0.40	$C_{50}H_{82}O_{21}$	$[M-H]^-$	841.5037,709.4591,353.1101	$C_{39}H_{65}O_{11}$−Xyl−GlurA	others			√
108[b]	24.63	781.4395	−1.16	$C_{41}H_{66}O_{14}$	$[M-H]^-$	437.7467	437−2H_2O−Rha−Glc	others	√	√	√
109[a]	24.75	829.4991	4.59	$C_{42}H_{72}O_{13}$	$[M-H+HCOOH]^-$	783.5156, 637.4358, 475.3782, 315.6922	20(*R*)−ginsenoside Rg2	PPT	√	√	√
110[b]	24.88	785.4344	1.25	$C_{40}H_{66}O_{15}$	$[M-H]^-$	503.6178,491.3740	OT−Xyl−Glc	OT		√	
111	25.25	955.4943	3.66	$C_{48}H_{76}O_{19}$	$[M-H]^-$	955.4951, 835.4491, 673.3979,631.3873, 455.3551	spinasaponin A 28−*O*−glucoside or isomer (OA−GlurA−2Glc)	OA	√	√	√
112	25.50	1087.5340	0.92	$C_{53}H_{84}O_{23}$	$[M-H]^-$	793.4341,731.4462,537.3897,455.3577	stipuleanoside R2 or isomer (OA−GlurA−2Glc−Xyl)	OA		√	
113	25.83	955.4943	3.66	$C_{48}H_{76}O_{19}$	$[M-H]^-$	955.4951, 673.3979,631.3873, 455.3551	spinasaponin A 28−*O*−glucoside or isomer (OA−GlurA−2Glc)	OA	√	√	√
114[a]	25.93	1153.6053	3.79	$C_{54}H_{92}O_{23}$	$[M-H+HCOOH]^-$	1107.5955,945.5462,783.4902,621.4394,459.3828,323.0978	ginsenoside Rb1	PPD	√	√	√
115	26.13	1087.5331	0.09	$C_{53}H_{84}O_{23}$	$[M-H]^-$	907.4690,793.4342,731.4348,613.3746,569.3914,537.3575,437.0645	stipuleanoside R2 or isomers (437−H_2O−GlurA−2Glc−Xyl)	OA			√
116	26.20	1101.5510	2.07	$C_{54}H_{86}O_{23}$	$[M-H]^-$	793.4433,631.401,613.3737,569.3852,455.3651	stipuleanoside R2 methyl ester or isomer (OA−GlurA−2Glc−Rha)	OA	√	√	√

No.	RT (min)	Observed *m/z*	ppm	Formula	Adducts	MS²	Identification	Type	M1	M2	M3
117	26.59	1087.5365	3.22	$C_{53}H_{84}O_{23}$	[M−H]⁻	1087.5338, 1049.3742, 967.4972, 907.4779, 793.4396, 749.4459, 731.4430, 659.4181, 613.3754, 587.3998, 533.7009, 523.3802, 455.3528	stipuleanoside R2 or isomer (OA−GlurA−2Glc−Xyl)	OA	√	√	√
118	26.61	955.4943	3.66	$C_{48}H_{76}O_{19}$	[M−H]⁻	793.4396,731.4393,569.3890,455.3532	spinasaponin A 28−*O*−glucoside or isomer (OA−GlurA−2Glc)	OA	√	√	√
119[b]	26.93	1191.5827	1.92	$C_{57}H_{92}O_{26}$	[M−H]⁻	1105.5786,943.5237,781.4800,619.4174,565.4881	$C_{36}H_{59}O_8$−3Glc−Mal	Mal	√	√	√
120	27.70	1087.5365	3.22	$C_{53}H_{84}O_{23}$	[M−H]⁻	955.4849,793.4424,731.4403,569.3860,455.3582	stipuleanoside R2 or isomer (OA−GlurA−2Glc−Xyl)	OA	√	√	√
121	28.11	955.4922	1.46	$C_{48}H_{76}O_{19}$	[M−H]⁻	793.4388,613.3752,569.3847,455.3505	chikusetsusaponin V or isomer (OA−GlurA−2Glc)	OA	√	√	√
122[b]	28.31	957.4982	6.76	$C_{44}H_{78}O_{22}$	[M−H]⁻	837.4577,795.4440,751.4542,615.3792,453.8991	453−Glc−H_2O−2Glc	others		√	
123	28.50	887.5041	3.68	$C_{44}H_{74}O_{15}$	[M−H+HCOOH]⁻	841.4969,799.4845,653.4289,635.4128,587.3991,491.3748	vinaginsenoside R1 or isomer (OT−Glc−Rha−Ace)	OT	√	√	√
124[a]	28.68	1123.5912	0.56	$C_{53}H_{90}O_{22}$	[M−H+HCOOH]⁻	1077.5852,915.5395,783.4875,573.4529,459.3829	ginsenoside Rc	PPD		√	√
125	28.82	1117.5469	2.93	$C_{54}H_{86}O_{24}$	[M−H]⁻	945.5430,793.4420,,621.4328	malonylfloralginsenoside Rd6 or isomer ($C_{36}H_{61}O_8$−2Glc−2Mal)	Mal	√	√	√
126[a]	29.01	1193.5994	2.85	$C_{57}H_{94}O_{26}$	[M−H]⁻	1107.5959,945.5441,783.4939,621.4388,459.3856	malonylginsenoside Rb1	PPD	√	√	√

No.	RT (min)	Observed *m/z*	ppm	Formula	Adducts	MS²	Identification	Type	M1	M2	M3
127	29.09	955.4909	0.10	$C_{48}H_{76}O_{19}$	$[M-H]^-$	793.4381,631.385,613.3755,455.3505	chikusetsusaponin V or isomer (OA−GlurA−2Glc)	OA			√
128[b]	29.87	825.4315	3.83	$C_{42}H_{66}O_{16}$	$[M-H]^-$	799.0272, 663.3744, 533.5886, 387.4493, 367. 1757	$C_{36}H_{55}O_{11}$−Glc	others	√	√	√
129	30.11	925.4820	1.90	$C_{47}H_{74}O_{18}$	$[M-H]^-$	793.4429,631.3885	elatoside A or isomer($C_{36}H_{56}O_{9}$−Glc−Xyl)	OA		√	√
130[b]	30.13	983.4557	4.11	$C_{48}H_{72}O_{21}$	$[M-H]^-$	855.4150,761.3575,435.0628	435−(Xyl−H_2O)−GlurA−(Glc−H_2O)−Ace	others	√	√	√
131	30.23	841.4961	0.71	$C_{44}H_{74}O_{15}$	$[M-H]^-$	737.3301,517.3927	notoginsenoside Rt or isomer ($C_{32}H_{54}O_{5}$−2Glc)	PPT	√	√	√
132[b]	30.45	983.4539	4.11	$C_{48}H_{72}O_{21}$	$[M-H]^-$	941.4283,761.3575,585.1025,435.0628	435−(Xyl−H_2O)−GlurA−(Glc−H_2O)−Ace	others		√	
133[b]	30.45	1005.4297	4.85	$C_{39}H_{74}O_{29}$	$[M-H]^-$	989.3463,963.3721,801.3270,471.0294	$C_{30}H_{57}O_{24}$−Glc−Ace	others			√
134	30.93	1087.5365	3.22	$C_{53}H_{84}O_{23}$	$[M-H]^-$	799.3416,731.4375,551.3736,455.3514	stipuleanoside R2 or isomer (OA−GlurA−2Glc−Xyl)	OA	√	√	√
135[a]	31.36	955.4948	3.66	$C_{48}H_{76}O_{19}$	$[M-H]^-$	793.4409,731.4394,569.3864,455.3537	ginsenoside Ro	OA	√	√	√
136	31.85	955.4943	3.66	$C_{48}H_{76}O_{19}$	$[M-H]^-$	793.4408,731.4396,569.3870,455.3551	chikusetsusaponin V or isomer (OA−GlurA−2Glc)	OA	√	√	√
137	32.46	955.4943	3.66	$C_{48}H_{76}O_{19}$	$[M-H]^-$	955.4927, 793.4404, 767.7324, 749.4456, 731. 4392, 631.3835, 613.3782, 587.3975, 569.3841 , 551.3757, 523.3826, 497.3741, 455.3523	chikusetsusaponin V or isomer (OA−GlurA−2Glc)	OA	√	√	√
138	32.55	1087.5365	3.22	$C_{53}H_{84}O_{23}$	$[M-H]^-$	925.4808,793.4388,731.4367,551.5736,455.35 43	stipuleanoside R2 or isomer (OA−GlurA−2Glc−Xyl)	OA	√	√	√

No.	RT (min)	Observed *m/z*	ppm	Formula	Adducts	MS^2	Identification	Type	M1	M2	M3
139	32.84	925.4794	3.78	$C_{47}H_{74}O_{18}$	[M−H]$^-$	805.4420, 673.3853, 631.3847, 613.3823, 569. 3855, 455.3503	chikusetsusaponin IV isomer (OA−GlurA−Glc−Xyl)	OA	√	√	√
140	33.16	955.4943	3.66	$C_{48}H_{76}O_{19}$	[M−H]$^-$	793.4414,731.4401,569.3857,455.3578	chikusetsusaponin V or isomer (OA−GlurA−2Glc)	OA	√	√	√
141[a]	33.86	1123.5934	2.60	$C_{53}H_{90}O_{22}$	[M−H+HCOOH]$^-$	1077.5892,945.5406,783.4912,621.4383,459.3 834	ginsenoside Rb3	PPD	√	√	√
142	34.56	955.4943	3.66	$C_{48}H_{76}O_{19}$	[M−H]$^-$	861.8203, 793.4421, 775.4396, 731.4360, 569. 3865, 537.3976, 523.3812, 455.3577	chikusetsusaponin V or isomer (OA−GlurA−2Glc)	OA	√	√	√
143	34.76	793.4414	4.28	$C_{42}H_{66}O_{14}$	[M−H]$^-$	631.3866,455.3560	chikusetsusaponin IVa isomer (OA−GlurA−Glc)	OA	√	√	√
144	34.84	925.4837	3.78	$C_{47}H_{74}O_{18}$	[M−H]$^-$	763.4303,613.3797,455.3543	pseudoginsenoside RT1 isomer (OA−Glc−Rha−Glc)	OA	√	√	√
145	35.29	955.4922	1.46	$C_{48}H_{76}O_{19}$	[M−H]$^-$	793.4388,631.3482,569.3844,455.3503	chikusetsusaponin V or isomer (OA−GlurA−2Glc)	OA		√	√
146	35.89	941.5123	0.85	$C_{48}H_{78}O_{18}$	[M−H]$^-$	761.4446,599.5458	calenduloside−B or isomer ($C_{42}H_{65}O_{12}$−H_2O−Glc)	OA		√	√
147	36.02	1193.5994	2.85	$C_{57}H_{94}O_{26}$	[M−H]$^-$	1107.5960945.5477,783.4881,621.4418,459.39 01	malonylginsenoside Rb1 isomer (PPD−4Glc−Mal)	PPD	√	√	√
148	36.23	955.4943	3.66	$C_{48}H_{76}O_{19}$	[M−H]$^-$	793.4421, 631.3846, 361.9299	spinasaponin A 28−*O*−glucoside or isomer ($C_{36}H_{56}O_9$−2Glc)	OA	√	√	√
149	36.51	955.4922	1.46	$C_{48}H_{76}O_{19}$	[M−H]$^-$	793.4413,731.4341,455.3577	chikusetsusaponin V or isomer (OA−GlurA−2Glc)	OA		√	

No.	RT (min)	Observed *m/z*	ppm	Formula	Adducts	MS[2]	Identification	Type	M1	M2	M3
150	36.53	855.4780	3.78	$C_{44}H_{72}O_{16}$	[M−H]⁻	669.5057,645.7305,517.3915	Notoginsenoside NL−D or isomer (PPT−Glc−Xyl−Mal)	PPT	√	√	√
151[a]	37.14	925.4837	3.78	$C_{47}H_{74}O_{18}$	[M−H]⁻	793.4387,719.4362,569.3865,455.3535	chikusetsusaponin IV	OA	√	√	√
152	37.36	793.4414	4.28	$C_{42}H_{66}O_{14}$	[M−H]⁻	793.4380, 631.3925, 569.3842, 513.3613, 455. 3511	chikusetsusaponin IVa isomer (OA−GlurA−Glc)	OA	√	√	√
153[a]	37.46	1163.5867	1.03	$C_{56}H_{92}O_{25}$	[M−H]⁻	1077.5868,945.5407,621.4347,459.3818	malonylginsenoside Rb2	Mal		√	
154	37.66	955.4943	3.66	$C_{48}H_{76}O_{19}$	[M−H]⁻	955.4909, 925.4753, 803.1356, 793.4431, 775. 4299, 763.4311, 731.4367, 659.4203, 631.3835 , 613.3803, 569.3847, 553.3814, 455.3579	spinasaponin A 28−*O*−glucoside or isomer (OA−GlurA−2Glc)	OA	√	√	√
155[a]	37.75	925.4837	3.78	$C_{47}H_{74}O_{18}$	[M−H]⁻	763.4295,701.4299,613.3763,569.3874,455.35 60	pseudoginsenoside RT1	OA	√	√	√
156	38.27	925.4837	3.78	$C_{47}H_{74}O_{18}$	[M−H]⁻	763.4347, 579.9621	pseudoginsenoside RT1 isomer (763−Glc)	OA	√	√	√
157	38.57	855.4780	3.85	$C_{44}H_{72}O_{16}$	[M−H]⁻	641.6438,555.7406,517.3897	Notoginsenoside NL−D or isomer (PPT−Glc−Xyl−Mal)	PPT	√	√	√
158[b]	39.43	1041.4940	2.19	$C_{51}H_{78}O_{22}$	[M−H]⁻	997.5052,835.4519,793.4368,773.4523,731.44 33,631.3835,613.3789,569.3863,455.3574	OA−GlurA−2Glc−Mal	OA	√	√	√
159	39.53	871.5061	0.00	$C_{44}H_{74}O_{14}$	[M−H+HCOOH]⁻	825.5018,783.4875,637.4367,475.3805	20(*S*)−6'−*O*−acetyl−ginsenosid e Rg2 or isomer (PPT−Glc−Rha−Ace)	PPT		√	√
160	39.59	975.5553	2.04	$C_{48}H_{82}O_{17}$	[M−H+HCOOH]⁻	929.5490,795.7238,701.4624,605.4441	vinaginsenoside R3 or isomer ($C_{36}H_{61}O_{7}$−2Glc)	others		√	√
161[a]	39.65	1163.5882	2.32	$C_{56}H_{92}O_{25}$	[M−H]⁻	1077.5848,1059.5700,783.4887,459.3817	malonylfloralginsenoside Rc1	PPD	√	√	√

No.	RT (min)	Observed *m/z*	ppm	Formula	Adducts	MS²	Identification	Type	M1	M2	M3
162[b]	40.66	1041.4940	2.19	$C_{51}H_{78}O_{22}$	[M−H]⁻	997.5058,837.4662,793.4323,713.4222,613.3748,455.3503	OA−GlurA−2Glc−Mal	OA	√	√	√
163	40.71	855.4780	3.85	$C_{44}H_{72}O_{16}$	[M−H]⁻	811.4827,683.0438,517.3951,475.3875,391.2876	Notoginsenoside NL−D or isomer (PPT−Glc−Xyl−Mal)	PPT	√	√	√
164[a]	41.75	991.5519	3.80	$C_{48}H_{82}O_{18}$	[M−H+HCOOH]⁻	945.5417,783.4929,621.4397,459.3900	ginsenoside Rd	PPD	√	√	√
165	43.00	793.4414	4.28	$C_{42}H_{66}O_{14}$	[M−H]⁻	793.4378, 673.4024, 631.3906, 569.3915, 455.3533	chikusetsusaponin IVa isomer (OA−GlurA−Glc)	OA	√	√	√
166[b]	43.11	861.4252	−4.08	$C_{41}H_{68}O_{19}$	[M−H]⁻	793.4425,653.3691,631.3667,515.9690	$C_{32}H_{55}O_{12}$−Glc−But	others			√
167[b]	43.13	831.4139	−6.09	$C_{44}H_{64}O_{15}$	[M−H]⁻	795.4429,633.3970,457.93602	457−GlurA−Glc−$2H_2O$	others			√
168[a]	43.40	793.4414	4.28	$C_{42}H_{66}O_{14}$	[M−H]⁻	631.3878,569.3875,455.3548	chikusetsusaponin IVa	OA	√	√	√
169[b]	43.60	843.4024	−0.17	$C_{40}H_{62}O_{16}$	[M−H+HCOOH]⁻	797.4476,651.8520,475.1381	PPT−GlurA−Xyl	PPT			√
170	43.86	793.4414	4.28	$C_{42}H_{66}O_{14}$	[M−H]⁻	631.3874,569.3866,455.3535	chikusetsusaponin IVa isomer (OA−GlurA−Glc)	OA	√	√	√
171[b]	44.91	1041.4940	2.19	$C_{51}H_{78}O_{22}$	[M−H]⁻	997.5014, 973.3751, 835.4477, 793.4343, 731.4389, 615.3838, 569.3854, 537.3930, 525.3930, 455.3504	OA−GlurA−2Glc−Mal	OA	√	√	√
172[b]	45.16	997.5040	2.11	$C_{50}H_{78}O_{20}$	[M−H]⁻	955.4968,937.4742,793.4326,613.3742,569.3843	$C_{42}H_{65}O_{14}$−Glc−Ace	OA	√	√	√
173	45.45	793.4414	4.28	$C_{42}H_{66}O_{14}$	[M−H]⁻	631.3853,569.3869,455.3503	zingibroside R1 isomer (OA−GlurA−Glc)	OA	√	√	√
174	45.76	991.5497	1.48	$C_{48}H_{82}O_{18}$	[M−H+HCOOH]⁻	945.5454,783.4887,621.4391,459.3841,323.1002	chikusetsusaponin FK7 or isomer (PPD−3Glc)	PPD		√	√
175	45.84	793.4414	4.28	$C_{42}H_{66}O_{14}$	[M−H]⁻	793.4419, 685.2588, 631.3799, 587.3975, 569.3858, 531.6812, 455.3607	zingibroside R1 isomer (OA−GlurA−Glc)	OA	√	√	√

No.	RT (min)	Observed *m/z*	ppm	Formula	Adducts	MS²	Identification	Type	M1	M2	M3
176[a]	45.85	1031.5462	2.91	$C_{51}H_{84}O_{21}$	[M−H]⁻	945.5473,783.4907,621.4404,459.3878	malonylfloralginsenoside Rd5	MAL	√	√	√
177[b]	46.17	831.4139	−6.09	$C_{44}H_{64}O_{15}$	[M−H]⁻	795.4413,793.4349,675.4051,633.3927,631.3841,571.3912,455.3504	$C_{35}H_{57}O_9$−Glc−2H_2O	OA			√
178	46.18	925.4837	3.78	$C_{47}H_{74}O_{18}$	[M−H]⁻	925.4853, 793.4420, 731.4365, 645.6688, 627.2022, 587.3987, 569.3883, 523.3942, 455.3560	chikusetsusaponin IV isomer (OA−GlurA−Glc−Xyl)	OA	√	√	√
179	46.39	955.4909	0.10	$C_{48}H_{76}O_{19}$	[M−H]⁻	793.4306	spinasaponin A 28−O−glucoside or isomer (793−Glc)	OA			√
180[b]	46.46	997.5040	2.11	$C_{50}H_{78}O_{20}$	[M−H]⁻	937.4834,793.4418,731.4371,569.3869,455.3505	OA−GlurA−2Glc−Ace	OA	√	√	√
181	46.56	1031.5427	−0.48	$C_{51}H_{84}O_{21}$	[M−H]⁻	997.4963,945.5332,783.4875,621.4414,569.3779,459.3531	malonylfloralginsenoside Rd1 or isomer (PPD−3Glc−Mal)	PPD			√
182[a]	46.66	991.5519	3.80	$C_{48}H_{82}O_{18}$	[M−H+HCOOH]⁻	945.5396,783.4961,621.4395,459.3756	Gypenoside XVII	PPD	√	√	√
183[b]	46.73	807.4373	−1.98	$C_{39}H_{68}O_{17}$	[M−H]⁻	627.3591	$C_{36}H_{51}O_9$−H_2O−Glc	others			√
184	46.83	1031.5462	2.91	$C_{51}H_{84}O_{21}$	[M−H]⁻	945.5390,783.4907,621.4365,459.3829	malonylfloralginsenoside Rd4 or isomer (PPD−3Glc−Mal)	PPD	√	√	√
185	47.00	955.4909	0.10	$C_{48}H_{76}O_{19}$	[M−H]⁻	793.4430	spinasaponin A 28−O−glucoside or isomer (793−Glc)	OA			√
186	47.03	1163.5867	1.04	$C_{56}H_{92}O_{25}$	[M−H]⁻	783.4778,621.4381	malonylfloralginsenoside Rc2 or isomers ($C_{36}H_{61}O_8$−2Glc−Xyl−Mal)	Mal		√	√
187[b]	47.12	997.5040	2.11	$C_{50}H_{78}O_{20}$	[M−H]⁻	937.4831,793.4391,731.4410,569.3849,455.3569	OA−GlurA−2Glc−Ace	OA	√	√	√

No.	RT (min)	Observed *m/z*	ppm	Formula	Adducts	MS^2	Identification	Type	M1	M2	M3
188	47.28	637.4352	4.86	$C_{36}H_{62}O_9$	[M−H]$^−$	475.3805, 347.8592	ginsenoside F1 or isomer (PPT−Glc)	PPT	√	√	√
189[b]	47.29	769.4408	2.98	$C_{40}H_{66}O_{14}$	[M−H]$^−$	637.4382,475.3813	PPT−Glc−Xyl	PPT	√	√	√
190[b]	47.75	1005.4850	−6.68	$C_{48}H_{78}O_{22}$	[M−H]$^−$	807.4614,609.3806,627.3943,537.3586,455.3534	OA−2Mal−2Glc−3H_2O	OA	√	√	√
191	47.85	1031.5462	2.91	$C_{51}H_{84}O_{21}$	[M−H]$^−$	945.5445, 783.4864, 643.6673, 595.6489, 573.4085, 553.0220, 535.5881, 459.3854	malonylfloralginsenoside Re1 or isomer (PPD−3Glc−Mal)	PPD	√	√	√
192[b]	47.94	997.5040	2.11	$C_{50}H_{78}O_{20}$	[M−H]$^−$	793.4399,731.4396,631.3879,569.3855,455.3539	OA−GlurA−2Glc−Ace	OA	√	√	√
193	47.95	1117.5469	2.93	$C_{54}H_{86}O_{24}$	[M−H]$^−$	945.5580,807.2544,783.4876,663.4469,621.4328,459.3829	malonylfloralginsenoside Rd6 or isomer (PPD−3Glc−2Mal)	PPD	√	√	√
194	48.38	975.5558	2.57	$C_{48}H_{82}O_{17}$	[M−H+HCOOH]$^−$	929.5487,731.4407,,605.4512,569.417,523.3801	vinaginsenoside R3 or isomer ($C_{36}H_{61}O_7$−2Glc)	others	√	√	√
195	48.38	939.4982	−2.66	$C_{48}H_{76}O_{18}$	[M−H]$^−$	793.4324,731.4369,569.3941,455.3515	bifinoside B or isomer (OA−Glc−GlurA−Rha)	OA	√	√	√
196	48.47	1031.5462	2.91	$C_{51}H_{84}O_{21}$	[M−H]$^−$	945.5457, 891.5329, 749.4744, 717.4537, 499.4052, 459.3896, 443.3519	malonylfloralginsenoside Re2 or isomer (PPD−3Glc−Mal)	PPD	√	√	√
197[b]	48.48	879.4416	3.66	$C_{45}H_{68}O_{17}$	[M−H]$^−$	835.4469,793.4352,775.4202,673.4001,631.3880,569.3909,455.3522	OA−GlurA−Glc−Mal	OA	√	√	√
198	48.68	1117.5441	0.45	$C_{54}H_{86}O_{24}$	[M−H]$^−$	945.5430,831.3983,621.4328	malonylfloralginsenoside Rd6 or isomer ($C_{36}H_{61}O_8$−2Glc−2Mal)	Mal		√	
199[a]	49.08	961.5402	2.67	$C_{47}H_{80}O_{17}$	[M−H+HCOOH]$^−$	621.4338,459.3829	notoginsenoside Fd	PPD	√	√	√
200[b]	49.23	1015.5507	2.34	$C_{51}H_{84}O_{20}$	[M−H]$^−$	929.5484,809.5067,767.4918,605.4442	$C_{31}H_{65}O_{13}$−2Glc−Mal	Mal	√	√	√

No.	RT (min)	Observed *m/z*	ppm	Formula	Adducts	MS²	Identification	Type	M1	M2	M3
201[b]	49.33	1001.5313	−1.89	$C_{50}H_{82}O_{20}$	$[M-H]^-$	915.5354,807.3641,783.4972,751.5302,641.6128,459.3722	PPD−2Glc−Xyl−Mal	PPD			√
202[a]	49.47	811.4859	1.30	$C_{42}H_{70}O_{12}$	$[M-H+HCOOH]^-$	765.4848,701.6191,619.4174,507.6964	ginsenoside F4	C-17 side-chain varied		√	√
203	49.64	943.5269	−0.32	$C_{48}H_{80}O_{18}$	$[M-H]^-$	765.4837,735.4626,603.4315,591.9684,525.1278,439.6161	ginsenoside Rh18 or isomer (591−2GlurA)	C-17 side-chain varied		√	√
204[b]	49.69	835.4489	0.42	$C_{44}H_{68}O_{15}$	$[M-H]^-$	631.3835,569.3902,455.3526	OA−GlurA−Glc−Ace	OA	√	√	√
205	49.70	721.4550	2.49	$C_{40}H_{66}O_{11}$	$[M-H]^-$	617.7764,479.9973,457.1973	dammar−12,24−dien−3*α*,6*β*, 15*α*−triol−3*α*−D−arabinopyranosyl−6*β*−L−arabinopyranoside or isomer (457−2Xyl)	others		√	√
206	49.72	679.4449	3.38	$C_{38}H_{64}O_{10}$	$[M-H]^-$	475.3805, 391.2847	3−*β*−acetoxyl ginsenoside F1 or isomer (PPT−Glc−Ace)	PPT	√	√	√
207[b]	49.74	809.4358	2.94	$C_{42}H_{66}O_{15}$	$[M-H]^-$	559.2375,475.3806	PPT−Glc−2Mal	PPT	√	√	√
208[b]	50.05	853.4611	−6.16	$C_{36}H_{72}O_{19}$	$[M-H+HCOOH]^-$	645.4001,469.4631,437.7406	469−GlurA−Glc	others		√	
209[b]	50.22	969.4915	0.83	$C_{45}H_{78}O_{22}$	$[M-H]^-$	793.4365,613.3731,549.3562,455.3507	OA−GlurA−Glc−GlurA	OA	√	√	√
210[b]	50.23	965.4812	0.18	$C_{42}H_{78}O_{24}$	$[M-H]^-$	793.4426,739.3943,645.5723,523.3790,481.8990,455.3577	OA−GlurA−Glc−2Mal	OA		√	
211[b]	50.24	871.4263	−1.04	$C_{44}H_{68}O_{15}$	$[M-H+Cl]^-$	835.4512,659.7577,631.3841,613.3754,569.3843,455.3503	OA−GlurA−Glc−Ace	OA		√	
212[b]	50.55	835.4516	3.65	$C_{44}H_{68}O_{15}$	$[M-H]^-$	775.4321,631.3751,569.3847,455.3539	OA−GlurA−Glc−Ace	OA	√	√	√
213	50.57	777.4447	2.18	$C_{42}H_{66}O_{13}$	$[M-H]^-$	567.8772,455.3516	bifinoside A or isomer (OA−GlurA−Rha)	OA		√	
214[a]	50.61	829.4991	4.59	$C_{42}H_{72}O_{13}$	$[M-H+HCOOH]^-$	621.4408,459.3829	ginsenoside F2	PPD	√	√	√

No.	RT (min)	Observed *m/z*	ppm	Formula	Adducts	MS²	Identification	Type	M1	M2	M3
215[b]	50.87	739.4301	3.63	$C_{39}H_{64}O_{13}$	$[M-H]^-$	529.9131, 475.3815, 437.8491	PPT−2Xyl	PPT	√	√	√
216	51.53	871.5055	−0.73	$C_{44}H_{74}O_{14}$	$[M-H+HCOOH]^-$	577.6422,401.7763	ginsenoside Rs3 or isomer	PPD			√
217	51.54	825.5014	0.97	$C_{44}H_{74}O_{14}$	$[M-H]^-$	783.4744,621.4331,601.9631	ginsenoside Rs3 or isomer ($C_{36}H_{61}O_8$−Glc−Ace)	PPD		√	
218[b]	51.57	807.4392	1.02	$C_{39}H_{68}O_{17}$	$[M-H]^-$	775.4289, 631.3840, 613.3665, 455.3503	OA−2GlurA	OA	√	√	√
219	51.63	925.4820	1.94	$C_{47}H_{74}O_{18}$	$[M-H]^-$	731.4353,631.3841,569.3852,455.3638	elatoside A or isomer (OA−GlurA−Glc−Xyl)	OA		√	√
220	51.90	811.4872	2.96	$C_{42}H_{70}O_{12}$	$[M-H+HCOOH]^-$	765.4809,603.4289	ginsenoside Rg6 or isomer ($C_{36}H_{60}O_7$−Glc)	C-17 side-chain varied	√	√	√
221[a]	52.12	793.4414	4.28	$C_{42}H_{66}O_{14}$	$[M-H]^-$	731.4393,613.3767,569.3865,455.3544	zingibroside R1	OA	√	√	√
222[b]	52.55	807.4392	0.37	$C_{39}H_{68}O_{17}$	$[M-H]^-$	793.4420,631.3882,455.3562	OA −2GlurA	OA	√	√	√
223	52.67	811.4872	2.96	$C_{42}H_{70}O_{12}$	$[M-H+HCOOH]^-$	765.4809,603.4289	ginsenoside Rg4 or isomer ($C_{36}H_{60}O_7$−Glc)	C-17 side-chain varied	√	√	√
224[b]	52.87	879.4416	3.66	$C_{45}H_{68}O_{17}$	$[M-H]^-$	835.4504, 793.4256,773.4488, 713.4325,, 613. 3776, 569.3922, 537.3584, 523.3827, 497.5445 , 483.3484, 455.3541	OA−GlurA−Glc−Mal	OA	√	√	√
225	52.97	925.4820	1.94	$C_{47}H_{74}O_{18}$	$[M-H]^-$	793.4344,731.4372,569.3843,455.3503	elatoside A or isomer (OA−GlurA−Glc−Xyl)	OA		√	√
226[b]	53.07	851.4821	2.64	$C_{45}H_{72}O_{15}$	$[M-H]^-$	807.4867,765.4807,603.4263	$C_{36}H_{59}O_7$−Glc−Mal	Mal	√	√	√
227[b]	53.13	865.4630	4.49	$C_{45}H_{70}O_{16}$	$[M-H]^-$	779.4586,717.4543,599.3948,509.3988	$C_{36}H_{55}O_7$−H_2O−Glc−Mal	Mal	√	√	√
228[b]	53.21	887.4420	−2.23	$C_{47}H_{68}O_{16}$	$[M-H]^-$	843.4527,801.4420,761.4516,621.3813	$C_{38}H_{53}O_7$−H_2O−Glc−Mal	Mal		√	√
229	53.41	763.4308	4.45	$C_{41}H_{64}O_{13}$	$[M-H]^-$	613.3760,569.3854,455.3577	pseudoginsenoside RP1 or isomer (OA−Glc−Rha)	OA	√	√	√

No.	RT (min)	Observed *m/z*	ppm	Formula	Adducts	MS^2	Identification	Type	M1	M2	M3
230	53.46	811.4840	−1.30	$C_{42}H_{70}O_{12}$	[M−H+HCOOH]$^-$	765.4261,457.3681	ginsenoside Rk1 or isomer ($C_{30}H_{50}O_3$−Glc−Rha)	C-17 side-chain varied			√
231	53.50	793.4414	4.28	$C_{42}H_{66}O_{14}$	[M−H]$^-$	713.4423, 675.4600, 631.3835,613.3756, 523.3 844, 455.3503	zingibroside R1 isomer (OA−GlurA−Glc)	OA	√	√	√
232	53.58	807.4917	2.08	$C_{44}H_{72}O_{13}$	[M−H]$^-$	603.4208,765.4753	ginsenoside Rs5 or isomer (603−Glc−Ace)	C-17 side-chain varied		√	√
233[b]	54.10	1175.6728	−6.05	$C_{56}H_{104}O_{25}$	[M−H]$^-$	995.6149,731.4463,569.3925,523.3865,437.34 73	437−3Xyl−Glc−(Glc−H_2O)	others		√	
234[b]	54.20	821.4703	1.57	$C_{44}H_{70}O_{14}$	[M−H]$^-$	735.4620,603.4294	$C_{36}H_{59}O_7$−Xyl−Mal	Mal		√	√
235[b]	54.26	857.4505	−4.72	$C_{43}H_{70}O_{17}$	[M−H]$^-$	821.4725,617.4218	$C_{33}H_{61}O_{10}$−Glc−Ace−2H_2O	OA		√	
236[b]	54.42	853.4611	−6.16	$C_{44}H_{70}O_{16}$	[M−H]$^-$	537.3610,455.3568	OA−GlurA−H_2O−Glc−Ace	OA		√	
237[b]	54.44	725.3846	−9.03	$C_{41}H_{58}O_{11}$	[M−H]$^-$	417.1505	417−Xyl−GlurA	others			√
238[b]	54.45	723.3839	4.20	$C_{34}H_{60}O_{16}$	[M−H]$^-$	415.1457	$C_{15}H_{27}O_{13}$−GlurA−Xyl	others	√	√	√
239[b]	54.45	843.4292	6.58	$C_{38}H_{68}O_{20}$	[M−H]$^-$	621.0086	621−(Glc−H_2O)−Ace	others			√
240[b]	54.50	1105.6680	−5.82	$C_{53}H_{102}O_{23}$	[M−H]$^-$	943.6084,793.4289,631.3889,455.3503	OA−GlurA−2Glc−H_2O−Xyl	OA		√	
241	54.62	763.4308	4.45	$C_{41}H_{64}O_{13}$	[M−H]$^-$	613.3736,569.3860,455.3519	pseudoginsenoside RP1 or isomer (OA−Glc−Rha)	OA	√	√	√
242[b]	54.69	663.4147	4.50	$C_{37}H_{60}O_{10}$	[M−H]$^-$	457.3608	457−H_2O−Rha−Ace	others	√	√	√
243	54.76	811.4840	−1.30	$C_{42}H_{70}O_{12}$	[M−H+HCOOH]$^-$	765.4837,605.4357	ginsenoside Rg5 or isomer (PPT−Glc−Xyl−Ace)	C-17 side-chain varied			√
244[b]	54.91	875.4532	4.00	$C_{38}H_{70}O_{19}$	[M−H+HCOOH]$^-$	667.0920,601.6053,491.5596	491−GlurA−Glc	others			√
245[b]	55.00	723.3839	4.20	$C_{34}H_{60}O_{16}$	[M−H]$^-$	415.1457	$C_{15}H_{27}O_{13}$−GlurA−Xyl	others	√	√	√
246[b]	55.37	669.3630	1.22	$C_{32}H_{58}O_{12}$	[M−H+Cl]$^-$	633.3922,457.3533	$C_{26}H_{50}O_6$−GlurA	others		√	
247[b]	55.37	699.3787	4.55	$C_{39}H_{56}O_{11}$	[M−H]$^-$	631.3863,455.3580	OA−GlurA−But	OA		√	√

No.	RT (min)	Observed *m/z*	ppm	Formula	Adducts	MS^2	Identification	Type	M1	M2	M3
248	55.40	811.4840	−1.30	$C_{42}H_{70}O_{12}$	$[M-H+HCOOH]^-$	765.4837,633.3839	ginsenoside Rz1 or isomer (633−Xyl)	C-17 side-chain varied			√
249[b]	55.42	1285.7584	−1.04	$C_{14}H_{15}O_{69}$	$[M-H]^-$	1109.7508	1109−GlurA	others			√
250[b]	55.46	667.3649	4.85	$C_{36}H_{56}O_9$	$[M-H+Cl]^-$	631.3873,613.3735,511.3438,455.3551	OA−GlurA	OA	√	√	√
251[b]	55.71	833.5225	1.26	$C_{50}H_{74}O_{10}$	$[M-H]^-$	569.6247	569−2Xyl	others		√	
252[b]	55.85	649.3457	2.68	$C_{30}H_{52}O_{12}$	$[M-H+HCOOH]^-$	603.8218,453.0642	453−H_2O−Xyl	others		√	
253	55.91	777.4455	3.13	$C_{42}H_{66}O_{13}$	$[M-H]^-$	597.3791,507.3859,437.3474	bifinoside A or isomer (437−H_2O−GlurA−Rha)	OA	√	√	√
254[b]	55.97	849.4658	1.27	$C_{45}H_{70}O_{15}$	$[M-H]^-$	805.4617,763.4643,745.4495,697.4399,603.4208,499.6071,469.3428	469−Xyl−Glc−Mal	Mal		√	
255[b]	56.03	805.4727	−2.07	$C_{44}H_{70}O_{15}$	$[M-H]^-$	763.4674,467.6741	467−H_2O−Rha−Xyl−Ace	others			√
256[b]	56.07	863.4451	1.29	$C_{45}H_{68}O_{16}$	$[M-H]^-$	819.4562,777.4412,615.3951,507.3836,437.3474	437−(GlurA−H_2O)−Rha−Mal	Mal		√	√
257[b]	56.25	631.3870	2.09	$C_{36}H_{56}O_9$	$[M-H]^-$	511.3688,455.3525	OA−GlurA	OA		√	√
258[b]	56.31	725.3945	−3.76	$C_{33}H_{60}O_{14}$	$[M-H+HCOOH]^-$	679.0198,499.7313	499−H_2O−Glc	others			√
259[b]	56.54	863.4451	1.90	$C_{45}H_{68}O_{16}$	$[M-H]^-$	819.45456,777.4339,513.6607,435.6497	435−2Glc−H_2O−Mal	Mal		√	√
260	56.79	665.4295	4.03	$C_{36}H_{60}O_8$	$[M-H+HCOOH]^-$	457.3681	ginsenoslaloside I or isomer ($C_{30}H_{50}O_3$−Glc)	C-17 side-chain varied	√	√	√
261[b]	56.85	661.3950	−1.88	$C_{37}H_{58}O_{10}$	$[M-H+HCOOH]^-$	645.1048,499.8089,453.3371	$C_{30}H_{45}O_3$−Glc	others			√
262[b]	56.99	801.4557	−3.27	$C_{48}H_{66}O_{10}$	$[M-H]^-$	765.2647,629.4236,603.4294	C36H59O7−Glc−2H_2O	others		√	
263[b]	57.19	809.4723	3.95	$C_{42}H_{68}O_{12}$	$[M-H+HCOOH]^-$	763.4620,601.4694	$C_{34}H_{65}O_8$−Glc	others	√	√	√
264[b]	57.61	849.4658	1.27	$C_{45}H_{70}O_{15}$	$[M-H]^-$	763.4655,601.4168	$C_{36}H_{57}O_7$−Glc−Mal	Mal		√	

No.	RT (min)	Observed *m/z*	ppm	Formula	Adducts	MS²	Identification	Type	M1	M2	M3
265[b]	57.94	1213.6557	−2.43	$C_{58}H_{102}O_{26}$	$[M-H]^-$	955.4890, 837.4663, 793.4415, 775.4299, 731.4371, 613.3751, 569.3850, 539.3713, 523.3797, 497.3664, 467.3606, 455.3532	OA−GlurA−2Glc−3Mal	OA	√	√	√
266[b]	59.81	747.4355	4.01	$C_{41}H_{64}O_{12}$	$[M-H]^-$	655.1988,567.3642	$C_{35}H_{51}O_6$−H_2O−Glc	others	√	√	√
267[b]	59.84	1051.6029	−2.79	$C_{52}H_{92}O_{21}$	$[M-H]^-$	757.0704, 631.3876, 613.3818, 595.3606, 523.3868, 497.3664, 455.3577	OA−GlurA−Glc−3Mal	OA	√	√	√
268[b]	60.13	769.4377	−1.05	$C_{34}H_{70}O_{16}$	$[M-H+Cl]^-$	733.4567,529.0912	529−Glc−Ace	others		√	
269[b]	64.79	975.5832	−7.28	$C_{50}H_{88}O_{18}$	$[M-H]^-$	679.6172,415.1456	415−3Xyl−(Rha−H_2O)	others		√	√
270[b]	64.94	977.5812	−3.92	$C_{52}H_{84}O_{14}$	$[M-H+HCOOH]^-$	755.3811	755−GlurA	others			√
271	66.68	859.4994	−1.46	$C_{43}H_{74}O_{14}$	$[M-H+HCOOH]^-$	561.2927	notoginsenoside SY4 or isomer	C-17 side-chain varied		√	√
272[b]	66.73	941.4568	−4.06	$C_{42}H_{72}O_{20}$	$[M-H+HCOOH]^-$	731.8690	731−H_2O−Rha	others			√

[a]: The components identified by comparison with the reference standards.

[b]: Unreported components from the *Panax* genus.

Table S4 Information of ten commercial chromatographic columns as the candidates for selecting the stationary phase in establishing the UHPLC/QTOF-MS approach.

Stationary Phase	Specification & Manufacturer	Separation Characteristics
**BEH C18**	2.1×100 mm, 1.7 μm； Waters	The column uses ethylene bridge hybrid particle technology, which widely used for various analytes. The triple-bonded alkyl column could take advantage of the ability of pH to exert a huge influence on the retention.
**BEH Shield RP18**	2.1×100 mm, 1.7 μm; Waters	In addition to the C18 chain, the column includes a hydrophilic carbamate functional group (polar embedded), which provides excellent peak shape for basic analytes. This column also offers other selectivity over linear alkyl columns.
**HSS T3**	2.1×100 mm, 1.8 μm; Waters	A triple-bond C18-bonding high-strength silica gel column with a relatively low carbon content (11%), enduring pure aqueous phase elution to enhance the retention of polar structures.
**CSH C18**	2.1×100 mm, 1.7 μm; Waters	The column is designed to increase sample loading and peak symmetry under low ionic strength mobile phase conditions, while still maintaining inherent mechanical and chemical stability of BEH particle technology.
**Zorbax Eclipse Plus C18**	2.1×100 mm, 1.8 μm; Agilent	The column can be used for excellent peak shape, while basic compounds that are very difficult to separate.
**Zorbax SB-Aq**	2.1×100 mm, 1.8 μm; Agilent	The column uses ultra-pure Rx-SIL silica gel, which could obtain excellent peak shapes for the analysis of many basic compounds.
**HSS C18 SB**	2.1×100 mm, 1.8 μm; Waters	An ultra-efficient and versatile C18 column is used to provide excellent peak shape for basic analytes and extremely long column life under low pH conditions.
**Cortecs UHPLC C18+**	2.1×100 mm, 1.6 μm； Waters	The column uses triple-bonded C18 technology of high-purity silica technology, which make it possible to use silica-based columns to develop high-resolution separations under high pH conditions.
**Zorbax Extend C18**	2.1×100 mm, 1.8 μm; Agilent	The column uses an innovative dual-coordination C18-C18 bonding technology, which make it possible to use silica-based columns to develop high-resolution separations under high pH conditions.
**ZORBAX SB-C18**	2.1×100 mm, 1.8 μm; Agilent	Silica gel with high purity and low acidity provides good peak shape for acidic, alkaline and neutral compounds. It can be used under high temperature and low pH condition and is stable without degradation, and compatible with common flows.
